# Global, regional, and national estimates of tuberculosis incidence and case detection among incarcerated individuals from 2000 to 2019: a systematic analysis

**DOI:** 10.1016/S2468-2667(23)00097-X

**Published:** 2023-06-29

**Authors:** Leonardo Martinez, Joshua L Warren, Anthony D Harries, Julio Croda, Marcos A Espinal, Rafael A López Olarte, Pedro Avedillo, Christian Lienhardt, Vineet Bhatia, Qiao Liu, Jeremiah Chakaya, Justin T Denholm, Yan Lin, Lisa Kawatsu, Limei Zhu, C Robert Horsburgh, Ted Cohen, Jason R Andrews

**Affiliations:** a Department of Epidemiology, School of Public Health, Boston University, Boston, MA, USA; b Department of Biostatistics, School of Public Health, Boston University, Boston, MA, USA; c Department of Global Health, School of Public Health, Boston University, Boston, MA, USA; d Department of Biostatistics, Yale School of Public Health, New Haven, CT, USA; e Department of Epidemiology of Microbial Diseases, Yale School of Public Health, New Haven, CT, USA; f Faculty of Infectious and Tropical Diseases, London School of Hygiene & Tropical Medicine, London, UK; g International Union Against Tuberculosis and Lung Disease, Paris, France; h School of Medicine, Federal University of Mato Grosso do Sul, Campo Grande, Brazil; i Oswaldo Cruz Foundation, Mato Grosso do Sul, Brazil; j Pan American Health Organization, Communicable Diseases and Environmental Determinants of Health, Washington, DC, USA; k Unité Mixte Internationale Trans VIHMI (UMI 233 IRD–U1175 INSERM, Université de Montpellier), Institut de Recherche pour le Développement, Montpellier, France; l Department of Communicable Diseases, World Health Organization Regional Office for South-East Asia, New Delhi, India; m Department of Chronic Communicable Disease, Center for Disease Control and Prevention of Jiangsu Province, Nanjing, China; n Department of Medicine, Therapeutics, Dermatology and Psychiatry, Kenyatta University, Nairobi, Kenya; o Department of Clinical Sciences, Liverpool School of Tropical Medicine, Liverpool, UK; p Victorian Tuberculosis Program, Melbourne Health, Melbourne, VIC, Australia; q Department of Microbiology and Immunology, Peter Doherty Institute for Infection and Immunity, University of Melbourne, Parkville, VIC, Australia; r Department of Epidemiology and Clinical Research, The Research Institute of Tuberculosis, Tokyo, Japan; s Division of Infectious Diseases & Geographic Medicine, School of Medicine, Stanford University, Stanford, CA, USA

## Abstract

**Background:**

People who are incarcerated are at high risk of developing tuberculosis. We aimed to estimate the annual global, regional, and national incidence of tuberculosis among incarcerated populations from 2000 to 2019.

**Methods:**

We collected and aggregated data for tuberculosis incidence and prevalence estimates among incarcerated individuals in published and unpublished literature, annual tuberculosis notifications among incarcerated individuals at the country level, and the annual number of incarcerated individuals at the country level. We developed a joint hierarchical Bayesian meta-regression framework to simultaneously model tuberculosis incidence, notifications, and prevalence from 2000 to 2019. Using this model, we estimated trends in absolute tuberculosis incidence and notifications, the incidence and notification rates, and the case detection ratio by year, country, region, and globally.

**Findings:**

In 2019, we estimated a total of 125 105 (95% credible interval [CrI] 93 736–165 318) incident tuberculosis cases among incarcerated individuals globally. The estimated incidence rate per 100 000 person-years overall was 1148 (95% CrI 860–1517) but varied greatly by WHO region, from 793 (95% CrI 430–1342) in the Eastern Mediterranean region to 2242 (1515–3216) in the African region. Global incidence per 100 000 person-years between 2000 and 2012 among incarcerated individuals decreased from 1884 (95% CrI 1394–2616) to 1205 (910–1615); however, from 2013 onwards, tuberculosis incidence per 100 000 person-years was stable, from 1183 (95% CrI 876–1596) in 2013 to 1148 (860–1517) in 2019. In 2019, the global case detection ratio was estimated to be 53% (95% CrI 42–64), the lowest over the study period.

**Interpretation:**

Our estimates suggest a high tuberculosis incidence rate among incarcerated individuals globally with large gaps in tuberculosis case detection. Tuberculosis in incarcerated populations must be addressed with interventions specifically tailored to improve diagnoses and prevent transmission as a part of the broader global tuberculosis control effort.

**Funding:**

National Institutes of Health.

## Introduction

More than 10 million people developed tuberculosis globally in 2021.^1^ Little improvement has been made in tuberculosis incidence in the past 20 years despite substantial investment in health resources.^2^ An important driver of this slow progress is the under-detection of tuberculosis cases in high-burden settings.^1^, ^3^ Approximately 30–40% of people who develop tuberculosis remain undiagnosed for long periods of time or are never diagnosed.^3^ These low detection rates have led to calls for targeted interventions to populations at high risk for tuberculosis who are often disproportionately underdiagnosed.^4^, ^5^, ^6^ Global and regional health organisations have focused their efforts on people living with HIV and close contacts of people with tuberculosis.^1^, ^7^ Further evidence is needed about the burden of tuberculosis in other groups at high risk to inform prioritisation of resources for targeted tuberculosis control.

A recent meta-analysis estimated that tuberculosis incidence among incarcerated individuals was ten times greater than that of surrounding communities.^8^ Additionally, millions of people enter and leave prison every year, and studies have shown that tuberculosis circulating in prisons might spill over into communities, amplifying transmission at the population level.^9^, ^10^, ^11^ Despite these circumstances, the epidemic of tuberculosis in prisons receives little attention in national and global tuberculosis reporting.^12^, ^13^, ^14^ WHO does not track or report tuberculosis cases in prisons, and most high-burden countries with tuberculosis do not routinely report these data publicly.^1^, ^2^, ^13^, ^14^, ^15^ Regional and global estimates of tuberculosis incidence among incarcerated populations are needed to identify gaps in case detection and treatment, as well as to allocate resources for addressing this epidemic.

**Research in context**

**Evidence before this study**

Two systematic reviews have pooled studies investigating tuberculosis incidence in prisons. Both systematic reviews found an overall high pooled incident tuberculosis rate in prisons among published studies but were limited in scope and global scale. The previous meta-analyses only contained study-level tuberculosis incidence data for about 35 countries. Additionally, the study-level data from these meta-analyses were biased towards high-income countries with Europe and the Americas overrepresented. Tuberculosis incidence in prisons has not previously been reported for most of the world, especially in high-burden, low-income settings. Neither of these previous meta-analyses estimated tuberculosis incidence globally, regionally, or nationally. In addition, tuberculosis notification data among incarcerated people have been reported for a few countries; however, these data have not been reported at a global scale. No studies have described aggregated notification data, adjusted for under-detection, or used these data to generate estimates for countries without reported data. Furthermore, trends in tuberculosis incidence over time in this population have not previously been presented.

**Added value of this study**

We collected several types of data for incarcerated individuals including tuberculosis incidence and prevalence estimates from published and unpublished studies, national tuberculosis notifications, and the number of incarcerated individuals from each country. Combining and harmonising these data, we developed a novel joint hierarchical Bayesian meta-regression approach to estimate several burden metrics at the country, regional, and global levels in locations that had no or very sparse data and for the first time including tuberculosis notification, incidence, and case detection ratios. Through this framework, we estimated that the incidence of tuberculosis globally among incarcerated individuals in 2019 was 125 105 (95% credible interval [CrI] 93 737–165 318), an annual rate of 1148 per 100 000 person-years (95% CrI 860–1517). Using this approach, we estimated that the number of tuberculosis cases among incarcerated individuals was substantially greater than that notified, with a case detection rate of 53% globally in 2019.

**Implications of all the available evidence**

The high tuberculosis incidence and low case detection rate in prisons globally indicate the need for greater attention and resources to address the disease burden in these settings. This compilation of national tuberculosis notifications from more than 150 countries indicates that these data are readily available from local officials and should be annually reported to regional and global health organisations as is currently done for other high-risk populations. Systematic data collection and urgent health policy action are needed to improve prompt diagnosis and care in prisons and other carceral facilities globally.

To address this knowledge gap, we aimed to estimate the global, regional, and national incidence of tuberculosis in incarcerated individuals in 2019. We also derived case detection ratios among incarcerated individuals and examined how tuberculosis incidence in incarcerated populations has changed from 2000 to 2019.

## Methods

### Study design

We estimated tuberculosis incidence, notification, and case-detection ratio among incarcerated populations regionally and globally. Incarcerated populations in our study referred to people in all carceral settings, and prisons and other correctional facilities are referred to as prisons.

The steps of this process comprised the following: compiling estimates of the tuberculosis incidence and prevalence from published and unpublished literature,^8^ collecting data for tuberculosis notifications among incarcerated populations, collecting the annual incarcerated population by country, and model building and analysis for joint prediction of each outcome of interest using a newly developed hierarchical Bayesian meta-regression framework.

### Data sources and collection

First, we did a systematic review and meta-analysis of the prevalence and incidence of tuberculosis among incarcerated people. The systematic review has been described previously.^8^ Briefly, we followed the PRISMA statement for the reporting of systematic reviews and meta-analyses. We searched MEDLINE, Embase, Web of Knowledge, and LILACS electronic databases from Jan 1, 1980, to Nov 15, 2020, without language restrictions, for studies reporting the tuberculosis incidence rate and prevalence among incarcerated populations. We used the following search terms, adapted for each database when appropriate: “Mycobacterium tuberculosis”, “tuberculosis”, “TB”, “incidence”, “prevalence”, “conversion”, “imprisonment”, “prison”, “inmate”, “transmission”, and “contact*” (appendix pp 8–11). Differing study designs were used for each outcome: cross-sectional surveys for tuberculosis prevalence and cohort studies for incidence. The systematic review^8^ is registered with PROSPERO, CRD42018104463.

Second, we collected national tuberculosis notifications in prisons. Currently, national tuberculosis notifications among incarcerated populations are not reported to WHO. Therefore, we contacted ministries of health, ministries of justice, and national tuberculosis programmes from 199 countries and territories (hereby referred to as countries) to solicit data for tuberculosis among incarcerated individuals. This list encompasses all WHO member countries in addition to several territories either not recognised by WHO or not officially designated as a country. In addition to contacting officials from each country directly, we collaborated with regional and global health organisations to communicate and work with country-level health officials to gather these data. These organisations were the International Union Against Tuberculosis and Lung Disease, the Pan American Health Organization, the WHO South-East Asian Regional Office, the WHO Eastern Mediterranean Regional Office, the European Centre for Disease Prevention and Control, the United Nations Office on Drugs and Crime, and the United Nations Office High Commissioner for Human Rights.

Specific information and a prespecified list of data were requested from each country. Required information for data inclusion was the raw number of incarcerated individuals diagnosed with tuberculosis in a specified year. We collected time trends of the annual notifications for each country when available. Additional data were age, sex, HIV co-infection status, the number of individuals incarcerated, and location (at the district, state, or city levels) of diagnosed cases. These additional data were reported by some countries but were not required for a country's inclusion in the analysis.

Lastly, we compiled national incarceration data from the Institute for Crime & Justice Policy Research or directly from governments.^16^ The total number of incarcerated individuals per year in each country was considered the average number in that given year. For each year, we requested data for the absolute number of incarcerated individuals, annual prison population rate, prison occupancy, number of penitentiary institutions, incarcerated individuals who were female, and pre-trial detainees. Occupancy was calculated as the number of incarcerated individuals divided by the capacity in those prisons. Additionally, we collected country-level metadata on economic (gross domestic product, World Bank income status, fragile states index, Human Development Index) and disease-specific characteristics (tuberculosis notifications and incidence, and HIV prevalence) from publicly available sources. We extracted WHO estimates of tuberculosis notification and incidence rates in the general population for each year and country.^1^

### Statistical analysis

A joint hierarchical Bayesian meta-regression framework was developed to model and predict tuberculosis incidence, notifications, and prevalence from all countries globally over time. Our primary outputs from the joint model were tuberculosis incidence and notifications. Incidence estimates among incarcerated individuals were taken from studies with active case finding interventions either as part of the study or as the routine standard of care. We constructed tuberculosis notification rates for each country by dividing the total number of diagnosed and reported cases by the total number of incarcerated individuals per year in that country.

In the meta-regression framework, true but unobserved cohort-level incidence was modelled as a function of predictors from the corresponding country and year. This included the notification rate, national incidence level, national income level (high, upper-middle, lower-middle, or low), and region-specific, country-specific, year-specific, and study-specific random effects. The random effects account for correlation between estimates from similar locations and times and the same study, and ensure accurate statistical inference for the predictions. In instances where country-level notifications among incarcerated individuals were known in a specific year, they were used directly as a predictor in the incidence regression model. Because we assumed that country-level notifications would be an important predictor of tuberculosis incidence, in situations where notifications were unknown, we developed a separate negative binomial regression model to estimate the missing predictor. This model used notifications at the year and country level as the outcome and the log of the number of incarcerated individuals as an offset term (ie, denominator). The same predictors included in the incidence regression model were used as well as global-specific, region-specific, country-specific, and year-specific random effects.

The incidence and notification regression models were fit jointly to correctly propagate uncertainty from the notification modelling to the final incidence predictions. We also included cohort-specific estimates of tuberculosis prevalence in the modelling framework to contribute additional information about incidence and notifications. We modelled prevalence as a function of incidence, notifications, and disease duration as detailed by Borgdorff's study,^17^ along with study-specific random effects. Prevalence was modelled jointly with incidence and notifications as a part of the meta-regression framework. We completed the model specification by assigning weakly informative prior distributions to the introduced parameters. Full details about the statistical model and prior distributions can be found in the appendix (pp 5–8).

The model was fit using a Markov chain Monte Carlo posterior sampling algorithm via the jags package within the R statistical computing software (version 4.2.1).^18^ Posterior inference is based on 30 000 samples collected from the joint posterior distribution of all model parameters (10 000 each from three independent chains) after removing the first 50 000 from each chain before convergence of the model and thinning the remaining 500 000 samples in each chain by a factor of 50 to reduce posterior autocorrelation. Convergence was assessed by calculating Geweke's diagnostic^19^ for key model parameters and effective sample sizes were calculated to ensure an adequate number of posterior samples had been collected before making inference. Using output from the model, we estimated tuberculosis incidence and notifications at the country level and regional level from 2000 to 2019. The case-detection ratio was estimated by dividing the notification rate by the incidence rate for each country. We report posterior medians and 95% highest posterior density credible intervals (CrIs) as point estimates and measures of uncertainty.

To validate the newly developed model with respect to predictive accuracy, we did a ten-fold cross-validation separately for incidence, notifications, and prevalence. We applied the model to the training datasets and used the output to predict the respective outcomes in the validation datasets exactly as we did with the full dataset analysis. We then compared the predicted and true outcomes graphically and calculated the proportion of times that the 95% CrIs contained the true value. These predicted values should fall within the credible intervals about 95% of the time if the model is performing well with respect to statistical inference.

### Role of the funding source

The funders had no role in study design, data collection, data analysis, data interpretation, writing of the report, decision to publish, or preparation of the manuscript.

## Results

**Figure 1:**
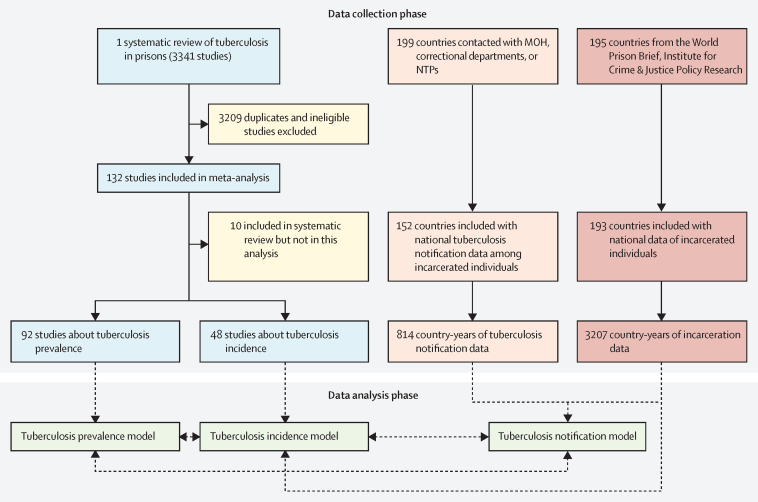
Flowchart of data collection and analysis Collection for each data type, statistical model structure, and connections between models is further described in the Article and appendix (pp 5–8). Seven countries were excluded from the analysis because of no tuberculosis or incarceration data: North Korea, Eritrea, occupied Palestinian territory, Serbia, Montenegro, Somalia, and Niue. Countries include all WHO member countries in addition to several territories either not recognised by WHO or not officially designated as a country. A full list of all countries with data can be found in the appendix (pp 13–16) as well as the countries not included. MOH=ministries of health. NTPs=national tuberculosis programmes.

We received national tuberculosis notifications among incarcerated individuals from 152 (76%) of 199 countries. Among included countries, many contributed multiple years of data; in total, 814 country-years of national notification data were collected (figure 1). We previously reported results from our systematic review and meta-analysis on tuberculosis in prisons.^8^ Briefly, we collected data for tuberculosis incidence from 48 studies and prevalence from 92 studies. Incarceration data were available for 193 countries with 3207 total country-years of data. In total, data were available from at least one data source from all countries except North Korea, Eritrea, Palestine, Serbia, Montenegro, Somalia, and Niue. These countries were excluded from our compilations, and, in total, we included 193 countries in our global estimations, representing more than 99% of the number of incarcerated individuals globally and 99% of global tuberculosis notifications.

We constructed the joint hierarchical Bayesian meta-regression model from the three sets of data to provide estimates for tuberculosis incidence and notifications among incarcerated individuals at a global, regional, and national level. In the validation analyses, model prediction accuracy for each outcome (tuberculosis incidence, notifications, and prevalence) was high (appendix p 24). The percentage of times the correct value was within the CrI for incidence, notifications, and prevalence was 92·7%, 94·6%, and 94·7%, respectively.

**Figure 2:**
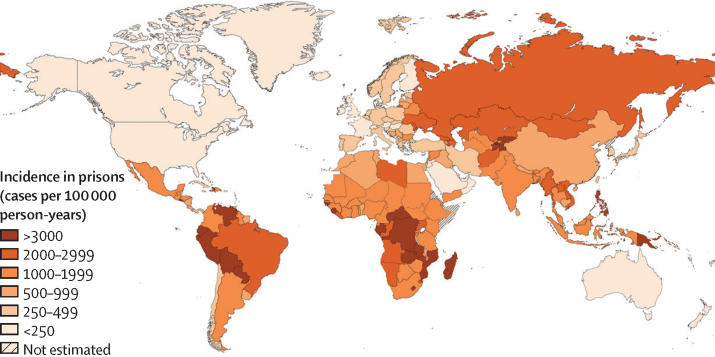
Estimated tuberculosis incidence in prisons (cases per 100 000 person-years) by country in 2019

In 2019, we estimated global tuberculosis incidence among incarcerated individuals to be 125 105 (95% CrI 93 736–165 318) and notifications to be 64 231 (56 516–72 788; table). Overall, the tuberculosis incidence rate per 100 000 person-years was 1148 (95% CrI 860–1517) but varied substantially by region (figure 2). From 2000 to 2012, global incidence per 100 000 person-years decreased consistently, from 1884 (95% CrI 1394–2616) to 1205 (910–1615; appendix p 29). From 2013 onwards, tuberculosis incidence per 100 000 person-years stayed generally stable, from 1183 (95% CrI 876–1596) in 2013 to 1148 (860–1517) in 2019. In 2019, the global case-detection ratio was 53% (95% CrI 42–64), the lowest of the study period (appendix p 31).

**Table:** Global and regional estimates of tuberculosis incidence and notifications among incarcerated individuals in 2019

	**Tuberculosis case notifications**		**Tuberculosis incidence**	
	Estimated number of notifications (95% CrI)	Rate per 100 000 person-years (95% CrI)	Estimated number of incident cases (95% CrI)	Rate per 100 000 person-years (95% CrI)
Africa	11 343(8107–15 148)	1195(854–1596)	21 280(14 377–30 522)	2242(1514–3216)
Americas	20 521(20 344–20 752)	543(538–548)	30 509(24 082–39 536)	807(637–1046)
Eastern Mediterranean	3058(1620–5241)	388(206–665)	6247(3386–10 579)	793(430–1342)
Europe	9408(9135–9821)	555(539–580)	20 058(12 581–32 816)	1184(743–1937)
South-East Asia	8536(4731–13 434)	641(355–1009)	18 894(10 573–30 790)	1419(794–2313)
Western Pacific	10 675(6098–16 469)	453(259–699)	25 994(14 631–42 233)	1104(621–1794)
Global	64 231(56 516–72 788)	589(519–668)	125 105(93 736–165 318)	1148(860–1517)

Data are estimated total number of notifications or incident cases (posterior medians) and the corresponding rate per 100 000 incarcerated person-years. For each estimate, we calculated the highest posterior density 95% CrI and rounded up to the nearest whole number. Total incidence estimates were calculated as the sum of tuberculosis incidence or notification values estimated for each WHO region. CrI=credible interval.

In 2019, the greatest absolute number of cases (30 509 [95% CrI 24 082–39 536]) and notifications (20 521 [20 344–20 752]) were estimated to occur in the Americas, followed by the Western Pacific region (25 994 cases [95% CrI 14 631–42 233] and 10 675 notifications [6098–16 469]) and African region (21 279 cases [14 377–30 521] and 11 343 notifications [8107–15 148]). The incidence rate per 100 000 person-years was highest in the African region (2242 [95% CrI 1515–3216]) and lowest in the Eastern Mediterranean region (793 [430–1342]). The case-detection ratio ranged from 42% (95% CrI 23–64) in the Western Pacific region to 68% (49–84) in the Americas region.

**Figure 3:**
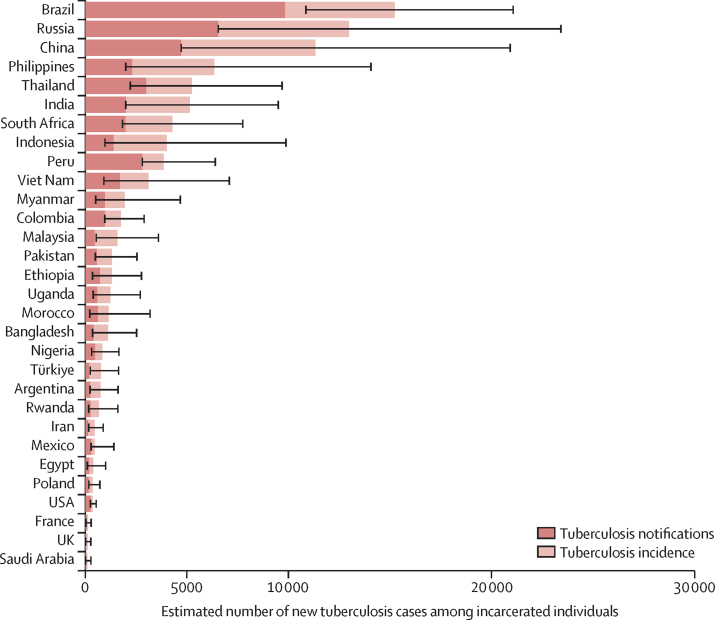
Estimated new tuberculosis cases and notifications among incarcerated individuals in 2019 for countries with the highest number of incarcerated people Error bars show the 95% highest posterior density credible intervals for incidence estimates. Estimated numbers are posterior medians.

In the 30 countries designated by WHO as high burden for tuberculosis, 87 258 incident cases (95% CrI 63 257–119 002) occurred among incarcerated individuals in 2019, representing 70% (95% CrI 64–75) of the global total in this population (appendix p 25). In the 30 countries with the most incarcerated individuals, 93 717 incident cases (95% CrI 69 117–127 669), representing 75% (95% CrI 70–80) of the global total, were estimated to occur. The five countries with the highest number of incident tuberculosis cases among incarcerated individuals in 2019 included Brazil (15 266 [95% CrI 10 876–21 099]), Russia (12 993 [6555–23 450]), China (11 347 [4733–20 956]), Philippines (6357 [1995–14 085]), and Thailand (5249 [2221–9710]; figure 3).

**Figure 4:**
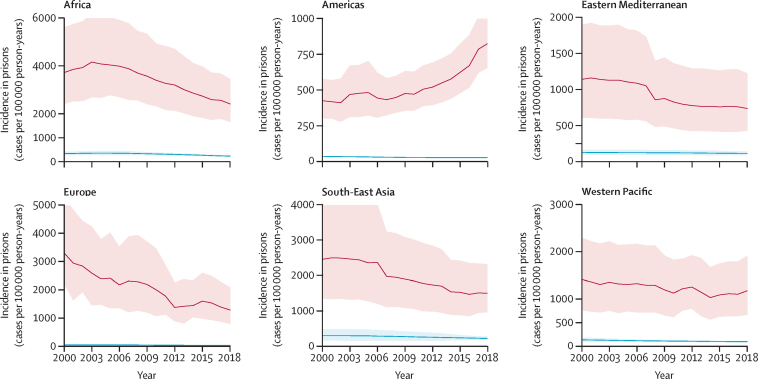
Trends in tuberculosis incidence estimates among incarcerated individuals compared with the general population for each WHO region from 2000 to 2019 The solid red line is modelled estimates (posterior medians) and the shaded bands are 95% highest posterior density credible intervals for tuberculosis cases in each WHO region. Blue lines represent tuberculosis incidence in the general population.

**Figure 5:**
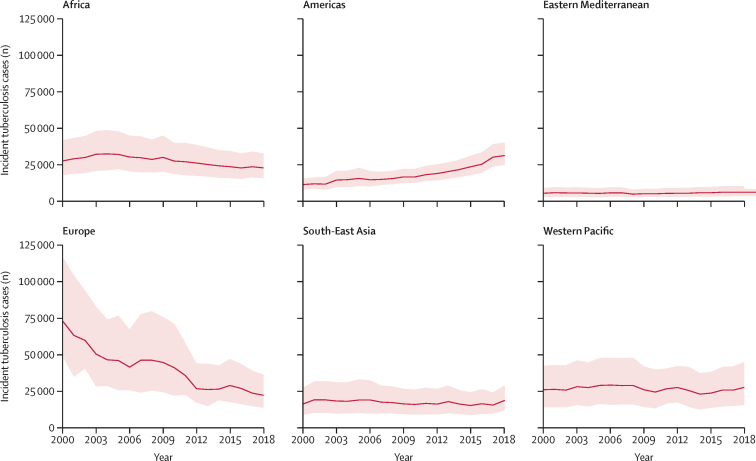
Trends in the absolute number of tuberculosis cases among incarcerated individuals for each WHO region from 2000 to 2019 The solid red line is modelled estimates (posterior medians) and the shaded bands are 95% highest posterior density credible intervals for tuberculosis cases in each WHO region.

From 2000 to 2019, the estimated tuberculosis incidence rate per 100 000 person-years increased 89% in the Americas from 426 (95% CrI 301–578) to 807 (637–1046) and the estimated absolute number of incident cases almost tripled (11 506 [95% CrI 8148–15 621] to 30 509 [24 082–39 536]; Figure 4, Figure 5). Tuberculosis incidence rates decreased in 2019 compared with 2000 in the African (3720 [95% CrI 2416–5610] *vs* 2242 [1515–3216]), European (3303 [2217–5269] *vs* 1184 [743–1937]), and South-East Asian (2452 [1344–4114] *vs* 1419 [794–2313]) regions. In the Western Pacific and Eastern Mediterranean regions, tuberculosis incidence among incarcerated individuals remained generally consistent over the study period.

Tuberculosis incidence in prisons was positively associated with both general population incidence (p<0·0001; adjusted R^2^ of 0·36) and overcrowding in prisons (p<0·0001; adjusted R^2^ of 0·16; appendix pp 40–41).

## Discussion

Using Bayesian meta-regression models integrating multiple sources of incarceration and tuberculosis data, we estimated that approximately 125 105 of the 11 million incarcerated individuals developed tuberculosis in 2019, an incidence rate of 1148 cases per 100 000 person-years. The estimated incidence rate in 2019 was greatest in the African region (2242 cases per 100 000 person-years); however, the Americas region, largely driven by Central and South America, had the largest estimated absolute number of tuberculosis cases (n=30 509). The estimated global casedetection ratio was low throughout the study period, reflecting major gaps in tuberculosis diagnosis in this population. Since 2013, tuberculosis incidence subsequently remained consistently between 1100 and 1200 cases per 100 000 person-years, suggesting that progress has stalled and current control measures are insufficient for mitigating transmission and disease in prisons.

The estimated global incidence of tuberculosis among incarcerated individuals is approximately nine times greater than the estimated incidence among all individuals (127 cases per 100 000 person-years).^1^ The elevated risk of tuberculosis among incarcerated populations exceeds that of diabetes (relative risk 1·5), alcohol use disorders (3·3), smoking (1·6), and undernourishment (3·2). Furthermore, we found a strong relationship between country-level tuberculosis incidence rates and overcrowding in prisons suggesting that decarceration might be one potential public health tool to combat the tuberculosis epidemic in prisons.

Important differences exist by WHO region over time. Incidence decreased in several regions (eg, European, African, and South-East Asian regions) and increased in the Americas region. Reasons for these trends are difficult to disentangle; however, several features might provide elucidation. Trends in Europe were largely influenced by prisons in Russia, which have successfully reduced tuberculosis incidence in the past decade due to targeted interventions.^20^, ^21^, ^22^ Importantly, estimated incidence in the African region was over 2200 cases per 100 000 person-years—almost double the global estimate—suggesting an urgent need for control measures and research on implementation of appropriate interventions. Reasons for the increases in tuberculosis burden in the Americas might be multifactorial. Mass incarceration has risen substantially in the Americas, possibly leading to increased crowding.^15^, ^16^ The strong association we found between crowding and tuberculosis incidence suggests this might be an important driver of the rising tuberculosis incidence in prisons in the Americas, especially in Central and South America.^8^, ^23^, ^24^ Although estimated incidence rates were high across WHO regions, heterogeneity was reported within regions as well. For example, in the Americas, incidence in South and Central American countries (both >1200 cases per 100 000 person-years) was considerably higher than those in North America (<50 incident cases per 100 000 person-years). Similarly, eastern Europe had a substantially higher incidence than western Europe.

Through a large systematic effort working with country-level officials, national and regional organisations, and non-governmental organisations, we were able to collect national notification data for tuberculosis in this hard-to-reach population. Global and regional health organisations currently focus efforts for tuberculosis reporting at the global level on certain populations at high risk (eg, people living with HIV and household contacts).^1^, ^25^ Incarcerated individuals have been excluded from these data compilations, either due to an assumption that these data are not available or a lack of prioritisation for this population.^1^, ^12^, ^26^, ^27^ Due to the scarcity of data, incarcerated populations have little representation in international tuberculosis guidelines and by national tuberculosis programmes.^1^, ^10^, ^12^, ^14^, ^26^ We were able to collect national tuberculosis notification data from more than 150 countries including more than 80% of WHO member states and 24 (80%) of 30 countries classified as high burden. Our collection efforts indicate that these data are accessible and retrievable from country-level officials, and that global health organisations should prioritise collecting data for tuberculosis among incarcerated individuals annually. Our analysis included data up to 2019, and tuberculosis notifications and incidence in prisons might have changed during the COVID-19 pandemic years. Further evaluation is necessary to understand the effect of the COVID-19 pandemic on tuberculosis within this population.

Increasing tuberculosis case detection is a major priority of global tuberculosis control. We found a low case-detection ratio in 2019 both globally and across WHO regions, ranging between 42% and 68%. These case-detection ratios are lower than for the general population and similar to ratios for paediatric tuberculosis, which is particularly difficult to diagnose.^1^, ^28^ Case-detection ratios were generally consistent over the study period and indicate that underdiagnosis is common in prisons globally. Our estimates of the case-detection ratio should be interpreted cautiously as they combine the output from both the tuberculosis incidence and notifications models instead of being modelled directly, potentially increasing uncertainty.

Our study has important strengths. First, we collected and combined a large array of global data for incarcerated individuals to inform our statistical models. Second, our study used a novel Bayesian meta-regression statistical approach, which allowed us to predict our various outcomes in locations with little coverage and quantify and report uncertainty. Our validation analyses found strong prediction accuracy and robust statistical inference (>90% of all withheld values were within the reported CrIs) giving increased confidence that our results are reliable. Although tuberculosis notifications among this population and incarceration-specific data (of which some data were absent) can be expanded, we expect additional and more refined data for tuberculosis among incarcerated individuals to become available in the future. This transparent and systematic approach that we propose in this study will allow these estimates to be improved over time in subsequent iterations.

These results should be interpreted within the context of several limitations. First, to estimate tuberculosis incidence among incarcerated individuals we made several assumptions that might lead to over or underestimation of the disease burden. We assumed that study-level results of tuberculosis in prisons are broadly representative in that country, which might overestimate incidence if published studies surveyed especially high tuberculosis-risk carceral settings. Empirical studies might have inherent biases in sampling and non-response that might affect overall outcomes. In particular, the CrIs reported here do not account for measurement, selection, and model specification bias. However, this approach is widely used by the Global Burden of Disease and other groups attempting to estimate regional and global disease burden.^29^, ^30^, ^31^ Second, our approach relies on an approximation of tuberculosis incidence from cohort studies in prisons with active case finding. Additionally, the types of active case finding and their implementation are likely to be heterogeneous across studies.^8^, ^22^ Third, tuberculosis notification data in prisons, as in the general population, might be subject to incomplete reporting. Reported notification rates were high across countries; however, this rate was much higher than for the general population. Further, expert consensus on diagnostic capacity is generally poor in prisons globally.^8^ Fourth, we were not able to provide incidence estimates between distinct carceral settings (ie, jails, immigrant detention centres, or facilities for juveniles), men and women separately, or groups at high risk within prisons (ie, people living with HIV) because of inadequate secondary data, both nationally and in the literature. Jails and other detention centres might differ in terms of environment, population characteristics, and health services, all of which can influence tuberculosis risk; further studies of tuberculosis incidence are needed in these settings. Women comprise about 7% of incarcerated individuals globally, a proportion that has been growing since 2000,^32^ but are underrepresented in studies of tuberculosis in prisons. Additionally, although prisons have been implicated in large multidrug-resistant tuberculosis outbreaks,^9^, ^33^ a paucity of systematic data precluded estimation of multidrug-resistant tuberculosis burden among incarcerated individuals. Finally, although we searched multiple electronic databases, the potential exists for missed articles.

To the best of our knowledge, our study represents the first global estimates of tuberculosis notification, incidence, and case-detection ratio among incarcerated populations. The high incidence rate globally and across regions, low case-detection rates, and consistency over time indicate that this population represents an important, underprioritised group for tuberculosis control. Continued failure to detect, treat, and prevent tuberculosis in prisons will result in unnecessary disease and deaths of many incarcerated individuals. Greater focus and resources for addressing the tuberculosis epidemic in prisons are needed to protect the health of incarcerated people and their communities.

## Data sharing

The data used for this analysis can be made available upon reasonable request from the corresponding author once all relevant substudies are reported and completed. The data dictionary can be made available upon request to the corresponding author.

## Declaration of interests

We declare no competing interests.
