# Correction to *Lancet Public Health* 2023; 8: e511–19

**DOI:** 10.1016/S2468-2667(23)00148-2

**Published:** 2023-07-13

*Martinez L, Warren JL, Harries AD, et al. Global, regional, and national estimates of tuberculosis incidence and case detection among incarcerated individuals from 2000 to 2019: a systematic analysis.* Lancet Public Health *2023; **8:** e511–19*—In this Article, the Declaration of interests section should have read “We declare no competing interests. RALO, PA, and MAE are staff members of the Pan American Health Organization. The authors alone are responsible for the views expressed in this publication, and they do not necessarily represent the decisions or policies of the Pan American Health Organization.” This correction has been made to the online version as of July 13, 2023.

