# Supplementary material for: Global, regional, and national estimates of tuberculosis incidence and case detection among incarcerated individuals from 2000 to 2019: a systematic analysis
Source: Lancet Public Health. 2023 Jun 29;8(7):e511–9. doi: 10.1016/S2468-2667(23)00097-X (PMC10323309; doi:10.1016/S2468-2667(23)00097-X)

THE LANCET
Public Health

Supplementary appendix

This appendix formed part of the original submission and has been peer reviewed. We post it as supplied by the authors.

Supplement to: Martinez L, Warren JL, Harries AD, et al. Global, regional, and national estimates of tuberculosis incidence and case detection among incarcerated individuals from 2000 to 2019: a systematic analysis. *Lancet Public Health* 2023; **8:** e511–19.

Supplementary Appendix.

Supplement to: Global, regional, and national estimates of tuberculosis incidence and case detection among incarcerated persons: a systematic analysis

Leonardo Martinez*, Joshua L. Warren*, Anthony D. Harries, Julio Croda, Marcos Espinal, Rafael A López-Olarte, Pedro Avedillo, Christian Lienhardt, Vineet Bhatia, Qiao Liu, Jeremiah Chakaya, Justin Denholm, Yan Lin, Lisa Kawatsu, Limei Zhu, C. Robert Horsburgh, Ted Cohen, Jason R. Andrews

*Contributed equally

Table of Contents.

Further Methodological Details

Joint Statistical Model for Incidence Rates, Notification Rates, and Prevalence Rates

Observed Data

Latent Processes

Random effects models

Prior Distributions

Posterior Inference

Imputation of incarceration persons by country

Table S1. Estimated absolute number of incident tuberculosis cases globally, 2000–2019

Table S2. Reporting of national tuberculosis notification among incarcerated persons by country and years of data.

Table S3. Estimates of tuberculosis incidence among incarcerated persons for all included countries in 2019.

Table S4. Case Detection Ratio Globally and within World Health Organization regions, 2019

Table S5. Model prediction accuracy for each outcome (prevalence, incidence, notifications) in cross-validation analyses

Table S6. Estimated tuberculosis cases and percent of global burden for differing groups of countries.

Table S7. Incidence and notification models and variables included

Table S8. Incidence rate ratios between prisons and the general population by WHO region and globally, 2019

Table S9. Estimated incident tuberculosis cases in prisons and the general population by WHO region and globally, 2019

Figure S1. Global tuberculosis incidence (cases per 100 thousand person-years) among incarcerated persons, 2000–2019

Figure S2. Absolute incident tuberculosis cases among incarcerated persons globally, 2000–2019

Figure S3. Global trends in case detection ratio among incarcerated persons, 2000–2019.

Figure S5. Tuberculosis incidence (cases per 100 thousand person-years) among incarcerated persons in Central, South, and North America, 2000–2019

Figure S6. Tuberculosis data availability on tuberculosis prevalence and/or incidence from the systematic review and meta-analysis and on national tuberculosis notifications among incarcerated persons.

Figure S7. Data availability on tuberculosis prevalence and/or incidence from the systematic review and meta-analysis, national tuberculosis notifications among incarcerated persons, and incarceration data.

Figure S8. Numbers of new tuberculosis cases (dark grey) and tuberculosis notifications (light grey) in the high tuberculosis burden countries.

Figure S9. Tuberculosis incidence per 100 thousand person-years and the absolute number of tuberculosis cases in each World Health Organization region, 2019.

Figure S10. Predicted values compared to observed tuberculosis incidence estimates.

Figure S11. Predicted values compared to observed tuberculosis notification estimates.

Figure S12. Predicted values compared to observed tuberculosis prevalence estimates.

Figure S13. The relationship between estimated tuberculosis incidence in prisons with tuberculosis incidence in the general population (figure S12; left hand panel) and capacity in prisons (figure S12; right hand panel).

Figure S14. Absolute number of incident tuberculosis cases in prisons and the general population over time, Eastern Mediterranean.

Figure S15. Absolute number of incident tuberculosis cases in prisons and the general population over time, Western Pacific.

Figure S16. Absolute number of incident tuberculosis cases in prisons and the general population over time, South-East Asia.

Figure S17. Absolute number of incident tuberculosis cases in prisons and the general population over time, Americas.

Figure S18. Absolute number of incident tuberculosis cases in prisons and the general population over time, Europe.

Further Methodological Details

Joint Statistical Model for Incidence Rates, Notification Rates, and Prevalence Rates:

Observed Data:

Model for Incidence Rates (Observed Data):

$$\hat{\theta}_{ijtkl} \sim \mathrm{N}\left(\theta_{ijtkl}, \hat{\sigma}^2_{ijtkl}\right);\ i = 1, \dots, r;\ j = 1, \dots, c_i;\ t = 1, \dots, s_{ij};\ k = 1, \dots, n_{ijt};\ l = 1, \dots, m_{ijtk}$$

- $\hat{\theta}_{ijtkl}$: Estimated log incidence rate from cohort *l,* within study *k,* within year *t*, within country *j,* within region *i*
- r: Total number of regions
- c_i: Total number of countries within region *i*
- s_{ij}: Total number of years from country *j* within region *i*
- n_{ijt}: Total number of studies in year *t*, within country *j,* within region *i*
- m_{ijtk}: Total number of cohorts from study *k,* in year *t*, within country *j,* within region *i*
- $\hat{\sigma}_{ijtkl}$: Standard error from cohort *l,* within study *k,* within year *t*, within country *j,* within region *i*
- θ_{ijtkl}: True but unobserved log incidence rate from cohort *l,* within study *k,* within year *t*, within country *j,* within region *i*

Model for Prevalence Rates (Observed Data):

$$\hat{\psi}_{ijtkl} \sim \mathrm{N}\left(\psi_{ijtkl}, \hat{\tau}^2_{ijtkl}\right)$$

- $\hat{\psi}_{ijtkl}$: Estimated log prevalence rate from cohort *l,* within study *k,* within year *t*, within country *j,* within region *i*
- $\hat{\tau}_{ijtkl}$: Standard error from cohort *l,* within study *k,* within year *t*, within country *j,* within region *i*
- ψ_{ijtkl}: True but unobserved log prevalence rate from cohort *l,* within study *k,* within year *t*, within country *j,* within region *i*

Model for Notification Rates (Observed Data):

$$Z_{ijt} \sim \text{Negative Binomial}\left(p_{ijt}, r\right),$$
$$p_{ijt} = \frac{r}{r + \lambda_{ijt}}$$
$$\ln\left(\lambda_{ijt}\right) = \mathrm{O}_{ijt} + \mathbf{x}_{ijt}^{\mathrm{T}} \boldsymbol{\beta}_z + \alpha_z + \mu_{zi} + \kappa_{zij}$$

- Z_{ijt}: Number of notifications in year *t*, from country *j,* within region *i*
- O_{ijt}: Offset amount (log of the estimated prison population; creates a rate interpretation) in year *t*, from country *j,* within region *i*
- $\mathbf{x}_{ijt}$: Vector of covariates (no intercept) that are complete across all regions/countries/years (i.e., no missingness) and explain variability in notification rates
- α_z: Global intercept
- μ_{zi}: Region-specific intercept

- κ_{zij}: Country-specific intercept

Latent Processes:

Model for True Incidence Rate (Latent Process):

$$\theta_{ijtkl} \sim \mathrm{N}\left(\theta_{ijt} + \pi_{\theta g(i,j,t,k)}, \sigma_\theta^2\right),$$

$$\theta_{ijt} = \max\left[\ln\left(\frac{Z_{ijt}}{\exp\{\mathrm{O}_{ijt}\}} + 1\mathrm{x}10^{-10}\right)\gamma_\theta + \mathbf{x}_{ijt}^{\mathrm{T}}\boldsymbol{\beta}_\theta + \alpha_\theta + \mu_{\theta i} + \kappa_{\theta ij} + \eta_{\theta ijt}, \ln\left(\frac{Z_{ijt}}{\exp\{\mathrm{O}_{ijt}\}} + 1\mathrm{x}10^{-10}\right)\right]$$

- θ_{ijt}: Log incidence rate in year *t*, from country *j*, within region *i*
    - max(.) function used because incidence rate must be bigger than or equal to notification rate
- $\ln\left(\frac{Z_{ijt}}{\exp\{\mathrm{O}_{ijt}\}} + 1\mathrm{x}10^{-10}\right)$: Log notification rate in year *t*, from country *j*, within region *i*
    - $1\mathrm{x}10^{-10}$ used in the code to avoid numerical problems
- $\mathbf{x}_{ijt}$: Same vector of covariates as in the notification rates model
- α_θ: Global intercept
- $\mu_{\theta i}$: Region-specific intercept
- $\kappa_{\theta ij}$: Country-specific intercept
- $\eta_{\theta ijt}$: Year-specific intercept
- $\pi_{\theta g(i,j,t,k)}$: Study-specific intercept; *g(.,.,.,.)* maps *i,j,t,k* to the larger study since some studies provide data from multiple countries and years (i.e., different *i,j,t,k* can map to the same study)
- σ_θ^2: Describes variability across cohorts within a study

Model for True Prevalence Rates (Latent Process):

$$\psi_{ijtkl} \sim \mathrm{N}\left(\ln(D) + \ln\left(\exp\{\theta_{ijt}\} - \frac{Z_{ijt}}{\exp\{\mathrm{O}_{ijt}\}}\right) + \pi_{\psi g(i,j,t,k)}, \sigma_\psi^2\right)$$

- Mean structure comes from equation 7 of Borgdorff (2004)
- D: Duration of disease; same parameter across all subscripts
- $\exp\{\theta_{ijt}\}$: Incidence rate in year *t*, from country *j*, within region *i*
- $\frac{Z_{ijt}}{\exp\{\mathrm{O}_{ijt}\}}$: Notification rate in year *t*, from country *j*, within region *i*
- $\pi_{\psi g(i,j,t,k)}$: Study-specific intercept; *g(.,.,.,.)* maps *i,j,t,k* to the larger study since some studies provide data from multiple countries and years (i.e., different *i,j,t,k* can map to the same study)
- σ_ψ^2: Describes variability across cohorts within a study

Random Effect Models:

Model for Region-Specific Effects:

$$\mu_{zi} \sim \mathrm{N}\left(0, \sigma_{\mu z}^2\right), \qquad \mu_{\theta i} \sim \mathrm{N}\left(0, \sigma_{\mu\theta}^2\right)$$

- $\sigma^2_{\mu z}, \sigma^2_{\mu\theta}$: Describes unexplained variability across regions for the incidence rate and notification rate outcomes

Model for Country-Specific Effects:

$$\kappa_{zij} \sim \mathrm{N}(0, \sigma^2_{\kappa z}), \qquad \kappa_{\theta ij} \sim \mathrm{N}(0, \sigma^2_{\kappa\theta})$$

- $\sigma^2_{\kappa z}, \sigma^2_{\kappa\theta}$: Describes unexplained variability across countries for the incidence rate and notification rate outcomes

Model for Year-Specific Effects:

$$\eta_{\theta ijt} \sim \mathrm{N}\left(0, \sigma^2_{\eta\theta}\right)$$

- $\sigma^2_{\eta\theta}$: Describes unexplained variability across regions for the incidence rate outcome

Model for Study-Specific Effects:

$$\pi_{\theta g(i,j,t,k)} \sim \mathrm{N}\left(0, \sigma^2_{\pi\theta}\right), \qquad \pi_{\psi g(i,j,t,k)} \sim \mathrm{N}\left(0, \sigma^2_{\pi\psi}\right)$$

- $\sigma^2_{\pi\theta}, \sigma^2_{\pi\psi}$: Describes unexplained variability across regions for the incidence rate and prevalence rate outcomes

---

Prior Distributions:

- $\beta_{zj}, \beta_{\theta j}, \gamma_\theta, \alpha_z, \alpha_\theta \sim \mathrm{N}(0, 100^2)$
- $\sigma^2_\theta, \sigma^2_\psi, \sigma^2_{\mu z}, \sigma^2_{\mu\theta}, \sigma^2_{\kappa z}, \sigma^2_{\kappa\theta}, \sigma^2_{\eta\theta}, \sigma^2_{\pi\theta}, \sigma^2_{\pi\psi} \sim \text{Inverse Gamma}(0.01, 0.01)$
- $r \sim \text{Gamma}(0.01, 0.01)$
- $D \sim \text{Uniform}(1,4)$

Posterior Inference:

Region *i*, country *j*, year *t*-specific estimates:

- Incidence rate: $\exp\{\theta_{ijt}\}$
- Prevalence rate: $\exp\{\psi_{ijt}\}$; $\psi_{ijt} = \ln(D) + \ln\left(\exp\{\theta_{ijt}\} - \frac{\lambda_{ijt}}{\exp\{\mathrm{O}_{ijt}\}}\right)$
- Notification rate: $\frac{Z_{ijt}}{\exp\{\mathrm{O}_{ijt}\}}$
- Case detection rate: (Notification rate)/(Incidence rate)
- Counts: (Incidence rate)*$\exp\{\mathrm{O}_{ijt}\}$, (Prevalence rate)*$\exp\{\mathrm{O}_{ijt}\}$, (Notification rate)*$\exp\{\mathrm{O}_{ijt}\}$, (Case detection rate)*$\exp\{\mathrm{O}_{ijt}\}$

Region *i*, year *t*-specific estimates:

- Incidence rate: $\frac{1}{\sum_{j=1}^{c_i} \exp\{\mathrm{O}_{ijt}\}} \sum_{j=1}^{c_i} \exp\{\mathrm{O}_{ijt}\} \exp\{\theta_{ijt}\}$

- Prevalence rate: $\frac{1}{\sum_{j=1}^{c_i} \exp\{O_{ijt}\}} \sum_{j=1}^{c_i} \exp\{O_{ijt}\}\exp\{\psi_{ijt}\}$
- Notification rate: $\frac{1}{\sum_{j=1}^{c_i} \exp\{O_{ijt}\}} \sum_{j=1}^{c_i} Z_{ijt}$
- Case detection rate: (Notification rate)/(Incidence rate)
- Counts: (Incidence rate)*$\left(\sum_{j=1}^{c_i} \exp\{O_{ijt}\}\right)$, (Prevalence rate)*$\left(\sum_{j=1}^{c_i} \exp\{O_{ijt}\}\right)$, (Notification rate)*$\left(\sum_{j=1}^{c_i} \exp\{O_{ijt}\}\right)$, (Case detection rate)*$\left(\sum_{j=1}^{c_i} \exp\{O_{ijt}\}\right)$

Global, year *t*-specific estimates:

- Incidence rate: $\frac{1}{\sum_{i=1}^{r}\sum_{j=1}^{c_i} \exp\{O_{ijt}\}} \sum_{i=1}^{r}\sum_{j=1}^{c_i} \exp\{O_{ijt}\}\exp\{\theta_{ijt}\}$
- Prevalence rate: $\frac{1}{\sum_{i=1}^{r}\sum_{j=1}^{c_i} \exp\{O_{ijt}\}} \sum_{i=1}^{r}\sum_{j=1}^{c_i} \exp\{O_{ijt}\}\exp\{\psi_{ijt}\}$
- Notification rate: $\frac{1}{\sum_{i=1}^{r}\sum_{j=1}^{c_i} \exp\{O_{ijt}\}} \sum_{i=1}^{r}\sum_{j=1}^{c_i} Z_{ijt}$
- Case detection rate: (Notification rate)/(Incidence rate)
- Counts: (Incidence rate)*$\left(\sum_{i=1}^{r}\sum_{j=1}^{c_i} \exp\{O_{ijt}\}\right)$, (Prevalence rate)*$\left(\sum_{i=1}^{r}\sum_{j=1}^{c_i} \exp\{O_{ijt}\}\right)$, (Notification rate)*$\left(\sum_{i=1}^{r}\sum_{j=1}^{c_i} \exp\{O_{ijt}\}\right)$, (Case detection rate)*$\left(\sum_{i=1}^{r}\sum_{j=1}^{c_i} \exp\{O_{ijt}\}\right)$

<u>Imputation of incarceration persons by country:</u>

A simple interpolation procedure was used to impute the number of incarcerated persons when this information was missing. Specifically, when the missing value had non-missing estimates from the same country in the years before and after the missing year, we averaged those values and rounded up to the nearest whole number (i.e., linear interpolation). If it only had a non-missing estimate in the year before or after, then we used that non-missing value as the estimate (i.e., constant interpolation). Finally, in cases where the missing value was surrounded by missingness in the years before and after, we iterated between the first two steps until all missing values were imputed.

Importantly, the incarcerated persons data were typically reported every other year in most countries. Most of the countries in our dataset (~55%) did not have two or more consecutive years of missing estimates at any point during 2000-2019. There were a small number of countries with more missingness, including Bhutan, Bosnia and Herzegovina, Equatorial Guinea, Guinea-Bissau, Laos, Oman, San Marino, South Sudan, and Syria. These countries have a generally low tuberculosis burden with a small influence on the regional and global level tuberculosis burden (among incarcerated populations and the general population. Therefore, the impact of missingness among these countries is low when estimating the macro-level burden estimates at the regional and global size.

<u>Search in Previously Published Systematic Review and Meta-analysis</u>

Pubmed – 1,094
Search completed August 6, 2020

(“tuberculosis"[MeSH Terms] OR "tuberculosis, multidrug-resistant"[MeSH Terms] OR “Mycobacterium tuberculosis”[MeSH] OR “Mycobacterium tuberculosis”[Text Word] OR “MDR -TB”[tw] OR “XDR -TB”[Text Word] OR “Mtb”[tw] OR (extensively[All Fields] AND ("drug resistance"[MeSH Terms] OR drug-resistant[Text Word]))) AND (detention[All Fields] OR jail[All Fields] OR gaol[All Fields] OR "correctional facility"[All Fields] OR "correctional facilities"[All Fields] OR incarcerat*[All Fields] OR imprisonment[All Fields] OR confinement[All Fields] OR inmates[All Fields] OR "prisons"[MeSH Terms] OR "prison*" [All Fields] OR Penitentiar*[tw] OR imprison*[tw] OR penal OR Criminals [MesH] OR "Concentration Camps" OR inmate*[tw] OR "Correctional setting"[tw] OR "Correctional settings"[tw] OR detain*[tw] OR detention*[tw] OR "Correctional Centre" [tw] OR "Correctional Centres"[tw] OR “compulsory drug detention” [tw] OR “compulsory drug treatment” OR “correction center” [tw] OR “laojiaosuo”[tw] OR “long-term detention” [tw] OR labor camp* [tw]) AND ("1980/01/01"[PDat] : "3000/12/31"[PDat])

Embase – 1,151
Search completed August 6, 2020

('tuberculosis'/de OR 'multidrug resistance' OR 'mycobacterium tuberculosis'/exp OR 'mdr -tb' OR 'xdr -tb' OR 'mtb' OR 'tb'/de) AND ('detention'/de OR jail OR gaol OR 'correctional facility' OR prison OR incarcerat* OR 'imprison*' OR detain* OR 'correction center' OR 'long-term detention' OR 'correctional settings' OR inmate*) AND (1980:py OR 1981:py OR 1982:py OR 1983:py OR 1984:py OR 1985:py OR 1986:py OR 1987:py OR 1988:py OR 1989:py OR 1990:py OR 1991:py OR 1992:py OR 1993:py OR 1994:py OR 1995:py OR 1996:py 7 OR 1997:py OR 1998:py OR 1999:py OR 2000:py OR 2001:py OR 2002:py OR 2003:py OR 2004:py OR 2005:py OR 2006:py OR 2007:py OR 2008:py OR 2009:py OR 2010:py OR 2011:py OR 2012:py OR 2013:py OR 2014:py OR 2015:py OR 2016:py OR 2017:py OR 2018:py OR 2019:py OR 2020:py)

Web of Science – 867
Search completed August 6, 2020 Search was restricted to articles after January 1, 1980 until the date of the search.

((TS=tuberculosis) OR TS=('mycobacterium tuberculosis') OR TS=(TB) OR TS=("MDR-TB") OR TS=("XDR-TB")) AND (TS=(detention) OR TS=(jail) OR TS=(gaol) OR TS=("correctional facility") OR TS=("correctional facilities") OR TS=(incarcerat*) OR TS=(imprisonment) OR TS=(confinement) OR TS=(inmates) OR TS=("prisons") OR TS=(imprison*) OR TS=(penal) OR TS=("Correctional Centres") OR TS=("Correctional Centre") OR TOPIC: (detain*) OR TS=(detention*) OR TS=(“correction center”) OR TS=("Concentration Camps") OR TS=(inmate*) OR TS=(“long-term detention”))

BIOSIS – 463
Search completed August 6, 2020
Search was restricted to articles after January 1, 1980 until the date of the search.

((TS=tuberculosis) OR TS=('mycobacterium tuberculosis') OR TS=(TB) OR TS=("MDR-TB") OR TS=("XDR-TB")) AND (TS=(detention) OR TS=(jail) OR TS=(gaol) OR TS=("correctional facility") OR TS=("correctional facilities") OR TS=(incarcerat*) OR TS=(imprisonment) OR TS=(confinement) OR TS=(inmates) OR TS=("prisons") OR TS=(imprison*) OR TS=(penal) OR TS=("Correctional Centres") OR TS=("Correctional Centre") OR TOPIC: (detain*) OR TS=(detention*) OR TS=(“correction center”) OR TS=("Concentration Camps") OR TS=(inmate*) OR TS=(“long-term detention”))

LILACS – 137
Search completed August 6, 2020 Search was restricted to articles after January 1, 1980 until the date of the search. 8

(tuberculosis [Words]) AND (prison [Words] or jail [Words])

INDMED – 3 Search completed

August 6, 2020 Search was restricted to articles after January 1, 1980 until the date of the search. (tuberculosis [Any Field]) AND (prison [Any Field] or jail [Any Field])

An additional search was done after from August 2020 to November 15, 2020. The results from this search can be seen below.

PubMed:

("tuberculosis"[MeSH Terms] OR "tuberculosis, multidrug-resistant"[MeSH Terms] OR "Mycobacterium tuberculosis"[MeSH] OR "Mycobacterium tuberculosis"[Text Word] OR "MDR -TB"[tw] OR "XDR -TB"[Text Word] OR "Mtb"[tw] OR (extensively[All Fields] AND ("drug resistance"[MeSH Terms] OR drugresistant[Text Word]))) AND (detention[All Fields] OR jail[All Fields] OR gaol[All Fields] OR "correctional facility"[All Fields] OR "correctional facilities"[All Fields] OR incarcerat*[All Fields] OR imprisonment[All Fields] OR confinement[All Fields] OR inmates[All Fields] OR "prisons"[MeSH Terms] OR "prison*" [All Fields] OR Penitentiar*[tw] OR imprison*[tw] OR penal OR Criminals [MesH] OR "Concentration Camps" OR inmate*[tw] OR "Correctional setting"[tw] OR "Correctional settings"[tw] OR detain*[tw] OR detention*[tw] OR "Correctional Centre" [tw] OR "Correctional Centres"[tw] OR "compulsory drug detention" [tw] OR "compulsory drug treatment" OR "correction center" [tw] OR "laojiaosuo"[tw] OR "longterm detention" [tw] OR labor camp* [tw]) AND ("2020/08/01"[PDat] : "3000/12/31"[PDat])

7 articles

Embase:

('tuberculosis'/de OR 'multidrug resistance' OR 'mycobacterium tuberculosis'/exp OR 'mdr -tb' OR 'xdr -tb' OR 'mtb' OR 'tb'/de) AND ('detention'/de OR jail OR gaol OR 'correctional facility' OR prison OR incarcerat* OR 'imprison*' OR detain* OR 'correction center' OR 'long-term detention' OR 'correctional settings' OR inmate*) AND (2020:py) AND ([1-8-2020]/sd) 25 articles Web of Science: ((TS=tuberculosis) OR TS=('mycobacterium tuberculosis') OR TS=(TB) OR TS=("MDR-TB") OR TS=("XDR-TB")) AND (TS=(detention) OR TS=(jail) OR TS=(gaol) OR TS=("correctional facility") OR TS=("correctional facilities") OR TS=(incarcerat*) OR TS=(imprisonment) OR TS=(confinement) OR TS=(inmates) OR TS=("prisons") OR TS=(imprison*) OR TS=(penal) OR TS=("Correctional Centres") OR TS=("Correctional Centre") OR TS=(detain*) OR TS=(detention*) OR TS=(“correction center”) OR TS=("Concentration Camps") OR TS=(inmate*) OR TS=(“long-term detention”)) Refined by: PUBLICATION YEARS: (2020)

8 articles

Biosis:

((TS=tuberculosis) OR TS=('mycobacterium tuberculosis') OR TS=(TB) OR TS=("MDR-TB") OR TS=("XDR-TB")) AND (TS=(detention) OR TS=(jail) OR TS=(gaol) OR TS=("correctional facility") OR TS=("correctional facilities") OR TS=(incarcerat*) OR TS=(imprisonment) OR TS=(confinement) OR TS=(inmates) OR TS=("prisons") OR TS=(imprison*) OR TS=(penal) OR TS=("Correctional Centres") OR TS=("Correctional Centre") OR TS=(detain*) OR TS=(detention*) OR TS=(“correction center”) OR

TS=("Concentration Camps") OR TS=(inmate*) OR TS=(“long-term detention”)) Refined by: PUBLICATION YEARS: (2020)

0 articles

For this additional search, Biosis and Web of Science we manually reviewed the articles from August 2020 onwards (as there was no simple way to classify the search by month).

Assessment of Study Quality from the Systematic Review and Meta-analysis

The quality of studies included in our meta-analysis were assessed with a modified version of the Newcastle-Ottawa scale. Studies were evaluated based on adequate participant selection (four points), comparability of studies based on design and analysis (one point), and adequate ascertainment of outcomes (three points). This scale awards a maximum of eight points. We defined studies that scored 66.6% or greater as high quality, those that scored 33.3–66.5% as moderate quality, and those that scored less than 33.3% as low quality.

Calculation of incidence if a cohort study reported cumulative incidence

If a cohort study reported cumulative incidence over a certain time span, we calculated the incidence by dividing the total number of incident cases by the time span of the follow-up. For example, if a cohort study reported 100 cumulative incident cases from 1,000 incarcerated persons over a 2 year follow-up time period, the incidence per 100,000 person-years would be 500.

Table S1. Estimated absolute number of incident tuberculosis cases globally, 2000–2019

Year	Absolute number of incident cases	Lower 95% CrI	Upper 95% CrI
2000	163,366	120,873	226,877
2001	159,018	114,798	223,506
2002	155,577	115,057	212,549
2003	152,933	110,489	211,251
2004	148,354	108,515	202,525
2005	150,688	110,296	208,167
2006	143,984	106,250	197,718
2007	146,681	106,482	202,319
2008	144,781	105,308	199,594
2009	142,218	103,839	194,003
2010	133,961	97,654	185,671
2011	132,646	99,130	178,578
2012	123,919	93,545	166,117
2013	123,243	91,302	166,308
2014	120,382	90,914	159,781
2015	123,756	92,973	165,535
2016	126,217	95,200	168,840
2017	127,623	97,070	167,873
2018	131,812	101,265	174,663
2019	125,105	93,737	165,318

Abbreviations. CrI, highest posterior density 95% credible intervals.

Table S2. Reporting of national tuberculosis notification among incarcerated persons by country and years of data.

Country	Years of data
Afghanistan	2017, 2018
Albania	2010-2012, 2014-2019
Andorra	2014-2019
Antigua and Barbuda	2008, 2010, 2012, 2014, 2016, 2017, 2019
Argentina	2014-2017, 2019
Armenia	2010, 2011, 2014-2019
Australia	2018
Austria	2012, 2014, 2016, 2018
Azerbaijan	2010, 2011, 2014-2017
Bahamas	2014-2019
Bangladesh	2018
Barbados	2019
Belarus	2010, 2015- 2019
Belgium	2010, 2014-2019
Belize	2008, 2010, 2012, 2014, 2019
Benin	2018
Bhutan	2018
Bolivia	2008-2010, 2012-2016, 2019
Bosnia and Herzegovina	2014, 2015
Botswana	2015, 2016, 2018
Brazil	2007-2019
Brunei Darussalam	2010-2018
Bulgaria	2010-2012, 2014-2019
Burkina Faso	2017, 2018
Cambodia	2014
Cameroon	2013
Canada	2000, 2001, 2003, 2005-2007
Central African Republic	2017
Chad	2018
Chile	2012-2017, 2019
China	2011, 2012
Colombia	2013, 2018, 2019
Comoros	2013, 2015, 2017, 2018
Costa Rica	2012-2019
Croatia	2014, 2016
Cuba	2019
Czech Republic	2010-2012, 2014-2019
Côte d'Ivoire	2010-2018
DR Congo	2016-2018
Denmark	2008, 2010, 2012, 2014-2017
Djibouti	2018
Dominica	2019

Dominican Republic	2011-2019
Ecuador	2014-2019
Egypt	2000-2001
El Salvador	2002-2019
Equatorial Guinea	2017, 2018
Estonia	2010-2012, 2014-2019
Fiji	2015-2018
Finland	2008-2010, 2012, 2015, 2016, 2018
France	2002, 2011, 2015-2018
Georgia	2010-2012, 2014-2019
Germany	2002
Ghana	2007-2013
Greece	2014-2018
Grenada	2019
Guatemala	2011, 2017-2019
Guinea	2017
Guyana	2001-2019
Haiti	2010-2018
Honduras	2007-2019
Hong Kong	2000-2005
Hungary	2011, 2012, 2014, 2016, 2018, 2019
Iceland	2011, 2014
India	2018
Indonesia	2015, 2018
Iran	2013, 2018
Ireland	2011, 2012, 2014-2019
Israel	2010-2012, 2014-2016
Jamaica	2015-2018
Japan	2000-2018
Jordan	2015-2018
Kazakhstan	2012, 2014-2019
Kenya	2017-2019
Kuwait	2018
Kyrgyzstan	2011, 2012, 2014-2019
Laos	2015-2018
Latvia	2010-2012, 2014-2017
Lebanon	2006, 2009, 2010, 2018
Lesotho	2017-2019
Liberia	2017
Libya	2018
Lithuania	2010-2012; 2014-2019
Luxembourg	2011, 2012, 2014-2019
Macedonia	2005, 2006, 2008, 2010-2012, 2014-2019
Malawi	2007, 2010, 2011-2018
Malaysia	2011-2015
Mali	2016-2018
Malta	2014-2019

Marshall Islands	2018
Mauritania	2017
Mexico	2012-2017, 2019
Moldova	2010-2012, 2014-2019
Monaco	2014, 2015
Mongolia	2001, 2002, 2004-2006, 2008, 2010, 2012-2016
Montenegro	2010, 2012, 2014, 2015, 2018, 2019
Mozambique	2015-2018
Myanmar	2018
Namibia	2015
Nepal	2015-2018
Netherlands	2010-2012, 2014-2019
New Zealand	2000, 2002, 2004, 2006, 2008, 2010, 2012, 2014-2016, 2018
Nicaragua	2014, 2016, 2018, 2019
Nigeria	2013-2016, 2018
Pakistan	2018
Panama	2010, 2012, 2014, 2016
Paraguay	2012, 2014-2019
Peru	2010-2019
Philippines	2014-2018
Poland	2010-2012, 2014-2019
Portugal	2011, 2012, 2014-2019
Puerto Rico	2019
Republic of Korea	2016-2018
Romania	2002, 2010-2019
Russia	2000, 2002, 2004, 2006, 2011, 2012, 2014-2019
Rwanda	2011-2015, 2017
Saint Lucia	2018, 2019
Samoa	2018
San Marino	2016-2018
Senegal	2013
Serbia	2011, 2012, 2014-2019
Seychelles	2005-2010, 2012, 2014, 2016, 2018
Slovakia	2010, 2011, 2014-2019
Slovenia	2014-2016, 2019
South Africa	2005, 2006, 2008, 2010
Spain	2002-2017
Suriname	2016-2019
Switzerland	2015, 2016
Taiwan	2005, 2006, 2008, 2010, 2012, 2014-2017
Tajikistan	2010-2012, 2014-2019
Thailand	2017, 2018
The Gambia	2012-2018
Timor-Leste	2018
Togo	2018
Tonga	2018
Trinidad and Tobago	2019

Tunisia	2018
Turkey	2010-2012, 2014-2019
Tuvalu	2009-2018
Uganda	2009
Ukraine	2010-2012, 2014-2019
United Arab Emirates	2018
United Kingdom	2011, 2012, 2014-2018
United States	2000, 2002, 2004-2006, 2008, 2010, 2012-2017, 2019
Uruguay	2004--2019
Uzbekistan	2011
Vanuatu	2018
Venezuela	2008--2017, 2019
Vietnam	2014-2017
Yemen	2018
Zambia	2008
Zimbabwe	2017

Table S3. Estimates (posterior medians) of tuberculosis incidence among incarcerated persons for all included countries in 2019.

Country	Incident tuberculosis cases per 100 thousand person-years	Lower 95% CrI	Upper 95% CrI
Afghanistan	2459	622	6455
Albania	454	114	1008
Algeria	737	139	1970
Andorra	0	0	2
Angola	2770	500	7485
Antigua and Barbuda	0	0	1
Argentina	690	214	1481
Armenia	477	136	1109
Australia	114	19	298
Austria	153	0	367
Azerbaijan	1730	960	3207
Bahamas	143	62	389
Bahrain	175	0	545
Bangladesh	1266	402	2866
Barbados	0	0	0
Belarus	682	185	1467
Belgium	325	182	604
Belize	493	261	1262
Benin	679	150	1673
Bhutan	1266	0	3210
Bolivia	2760	1796	5545
Bosnia and Herzegovina	435	0	1202
Botswana	1482	456	3225
Brazil	2135	1521	2951
Brunei Darussalam	426	0	1251
Bulgaria	480	109	1069
Burkina Faso	1170	376	2528

Burundi	1712	313	4650
Cambodia	2347	475	5675
Cameroon	1307	502	2499
Canada	25	0	88
Cape Verde	572	0	1616
Central African Republic	10533	2996	24142
Chad	1919	489	4692
Chile	290	127	598
China	671	280	1240
Colombia	1465	804	2421
Comoros	2	0	1623
Congo (Brazzaville)	2891	458	7858
Costa Rica	343	228	917
Croatia	166	0	432
Cuba	227	122	629
Cyprus	0	0	492
Czech Republic	132	37	279
Côte d'Ivoire	1870	626	4207
DR Congo	7917	2832	17478
Denmark	129	0	340
Djibouti	2081	0	5272
Dominica	0	0	2
Dominican Republic	1348	1348	3040
Ecuador	2492	2364	4865
Egypt	331	81	836
El Salvador	3336	3336	4430
Equatorial Guinea	1781	372	4244
Estonia	251	81	529
Eswatini	2826	523	7671
Ethiopia	1185	322	2519
Fiji	590	0	1576
Finland	119	0	334
France	199	59	411

Gabon	3459	492	9430
Georgia	1192	522	2388
Germany	250	72	542
Ghana	1000	247	2287
Greece	291	92	633
Greenland	1587	0	5556
Grenada	0	0	1
Guatemala	972	972	2303
Guinea	2484	799	5375
Guinea-Bissau	4309	671	12594
Guyana	1213	372	2603
Haiti	3000	737	7856
Honduras	973	973	2062
Hong Kong	536	108	1414
Hungary	185	66	385
Iceland	0	0	943
India	1076	417	1988
Indonesia	1522	368	3758
Iran	244	89	470
Iraq	649	117	1830
Ireland	105	0	307
Israel	178	59	351
Italy	194	45	448
Jamaica	148	0	471
Japan	240	68	511
Jordan	99	0	293
Kazakhstan	1752	784	3582
Kenya	1912	935	3953
Kiribati	3692	0	10853
Kuwait	408	67	1046
Kyrgyzstan	2800	1617	5560
Laos	2064	557	4766
Latvia	714	203	1560

Lebanon	405	88	1021
Lesotho	3676	2073	7723
Liberia	3111	740	7507
Libya	2516	625	5687
Lithuania	653	293	1389
Luxembourg	331	182	661
Macedonia	344	94	701
Madagascar	3094	641	8603
Malawi	1643	763	2793
Malaysia	2208	753	5010
Maldives	326	0	1038
Mali	918	314	1845
Marshall Islands	4	0	11585
Mauritania	1136	216	2714
Mauritius	133	0	425
Mexico	215	135	656
Micronesia	1264	216	3388
Monaco	0	0	1
Mongolia	2367	617	5111
Montenegro	451	116	1012
Morocco	1348	261	3763
Mozambique	5347	1610	11769
Namibia	2904	740	6908
Nauru	0	0	7143
Nepal	939	201	2382
Netherlands	274	173	437
New Zealand	121	0	321
Niger	1427	279	3886
Nigeria	1150	435	2271
Norway	113	0	347
Oman	128	0	445
Pakistan	1590	600	3102
Panama	399	82	1064

Papua New Guinea	3853	640	10717
Paraguay	3010	3010	5444
Peru	4194	3052	6956
Philippines	3839	1205	8507
Portugal	487	306	884
Puerto Rico	0	0	0
Qatar	389	0	1271
Republic of Korea	408	81	1099
Romania	857	531	1704
Rwanda	930	241	2266
Saint Kitts and Nevis	0	0	455
Saint Lucia	0	0	1
Saint Vincent and the Grenadines	0	0	578
Samoa	1	0	989
Sao Tome and Principe	1102	0	3286
Saudi Arabia	160	21	464
Senegal	1636	457	3696
Serbia	256	60	626
Seychelles	0	0	588
Singapore	413	55	1219
Slovakia	158	57	326
Slovenia	0	0	2
Solomon Islands	841	0	2557
South Africa	2903	1240	5250
Spain	317	117	631
Sri Lanka	579	83	1623
Sudan	1562	250	4611
Suriname	668	200	1423
Sweden	170	0	447
Switzerland	151	0	376
Syria	609	101	1851
Taiwan	450	100	1184
Tajikistan	3007	1325	5934

Tanzania	2050	415	5497
Thailand	1479	626	2736
The Gambia	2028	0	4857
Timor-Leste	2429	0	6227
Togo	822	168	2185
Tonga	0	0	803
Trinidad and Tobago	342	342	894
Tunisia	454	92	1176
Turkey	267	84	565
Turkmenistan	1123	208	2928
Tuvalu	2	0	11325
Uganda	2071	653	4514
Ukraine	2289	1315	4499
United Arab Emirates	70	0	193
United Kingdom	144	32	342
United States	17	12	26
Uruguay	1011	941	2261
Uzbekistan	1286	284	3045
Vanuatu	1	0	1757
Venezuela	4419	4419	6493
Vietnam	2480	730	5647
Yemen	900	165	2436
Zambia	3530	1197	7618
Zimbabwe	1599	400	3720

Abbreviations. CrI, highest posterior density 95% credible intervals.

Table S4. Case Detection Ratio Globally and within World Health Organization regions, 2019

WHO Region	Case detection ratio (%)	Low 95% CrI	High 95% CrI
Africa	52.1	37.3	67.2
Americas	67.9	49.3	84.0
Eastern Mediterranean	45.0	25.6	66.7
Europe	42.5	27.3	58.0
South-East Asia	45.4	25.4	67.0
Western Pacific	42.3	23.2	64.2
Global	52.8	41.8	63.7

Table S5. Model statistical inference performance for each outcome (incidence, notifications, prevalence) in cross-validation analyses

Model outcome	True value within the Credible Intervals (%)
Incidence	94.1
Notifications	91.4
Prevalence	94.9

Table S6. Estimated tuberculosis cases and percent of global burden for differing groups of countries in 2019.

Category	N, countries	N, tuberculosis cases (95% CrI)	Percent of Global Burden (95% CrI)
WHO 'High-Burden' countries	29	87,258 (63,257–119,002)	70 (64–75)
Countries with the greatest number of incarcerated persons	30	93,717 (69,117–127,669)	75 (70–80)
Countries overlapping on both lists	14	78,129 (56,478–108,318)	63 (56–69)
4 countries with most incarcerated persons (India, China, Brazil, Russia)	4	45,966 (31,670–65,878)	37 (29–45)

Table S7. Incidence and notification models and variables included

Model and variables included	Estimate	low 95% CI	high 95% CI
**Incidence Model**			
National tuberculosis Notifications (Prisons)	0.42	0.25	0.58
National tuberculosis incidence (General population)	0.23	0.01	0.46
National income level			
Lower-Middle vs. Low Income:	-0.37	-0.83	0.08
Upper-Middle vs. Low Income:	-0.44	-0.97	0.08
High vs. Low Income:	-1.42	-2.19	-0.68
**Notifications Model**			
National tuberculosis incidence (General population)	0.73	0.53	0.92
Income			
Lower-Middle vs. Low Income:	-0.52	-0.80	-0.24
Upper-Middle vs. Low Income:	-1.06	-1.39	-0.72
High vs. Low Income:	-1.43	-1.82	-1.05

Both outcomes and predictors in this table are on log-scales. A positive/negative estimate indicates a relationship between X and Y generally and also indicates that that particular variable was important in estimating/predicting outcomes in areas without data.

Table S8. Incidence rate ratios between prisons and the general population by WHO region and globally, 2019

Region	Incidence Ratio Ratios (rate in prisons/general population)	
	Notifications	Incidence
Africa	9.3	8.8
Americas	21.7	29.0
Eastern Mediterranean	6.5	6.9
Europe	22.2	45.1
South-East Asia	5.3	6.5
Western Pacific	5.7	11.7
Global	6.2	8.9

Table S9. Estimated incident tuberculosis cases in prisons and the general population by WHO region and globally, 2019

**Region**	**Estimated total incident cases**	
	**Prisons**	**Total**
Africa	21,280	2,448,720
Americas	30,509	259,491
Eastern Mediterranean	6,247	812,753
Europe	20,058	225,942
South-East Asia	18,894	4,321,106
Western Pacific	25,994	1,774,006
Global	125,105	9,834,895

Figure S1. Global tuberculosis incidence (cases per 100 thousand person-years) among incarcerated persons, 2000–2019

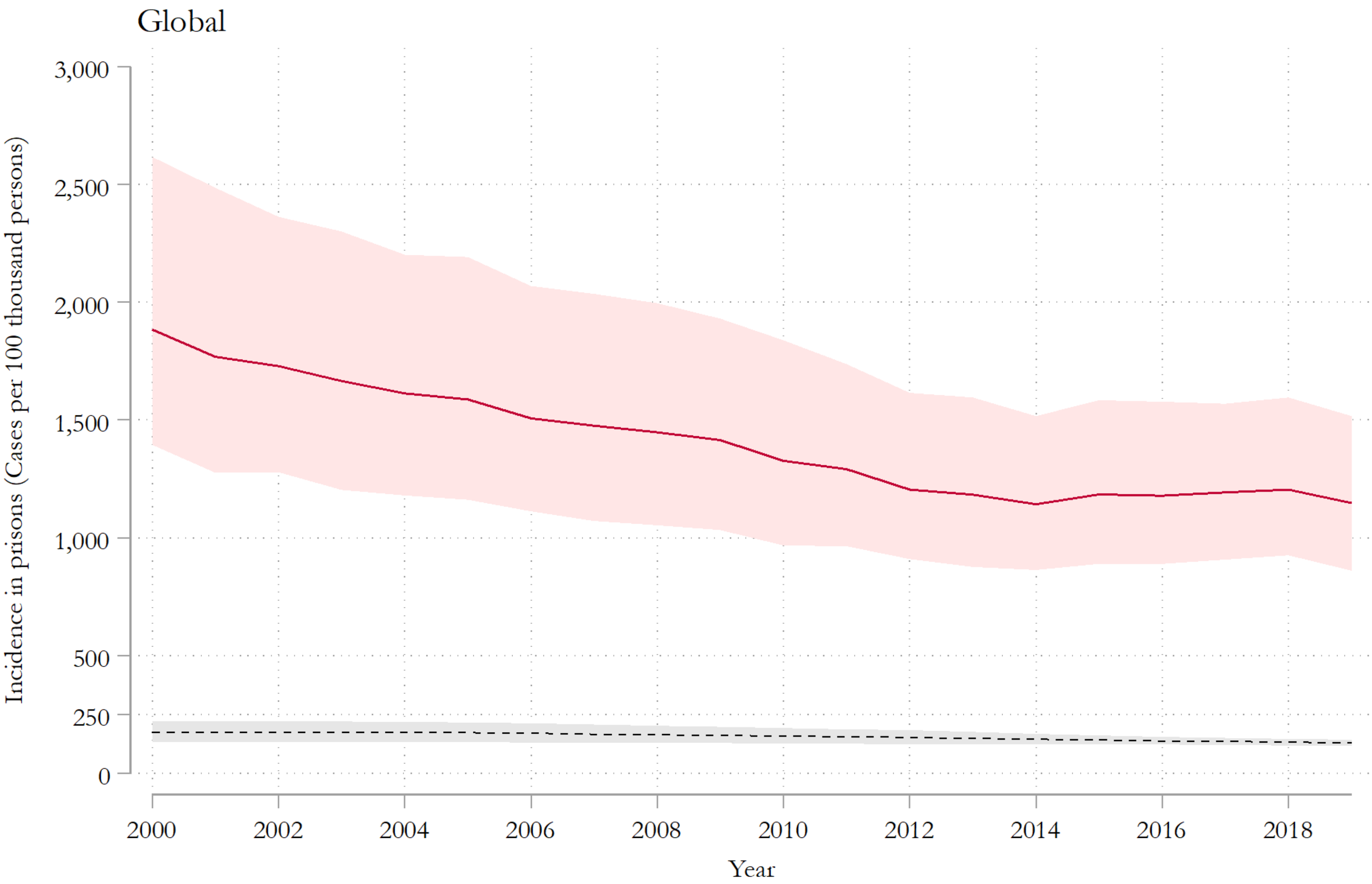

Solid red line and shaded band represent modelled point estimates (posterior medians) and 95% credible intervals for tuberculosis cases globally. Dashed line represents tuberculosis incidence in the general population globally.

Figure S2. Absolute incident tuberculosis cases among incarcerated persons globally, 2000–2019

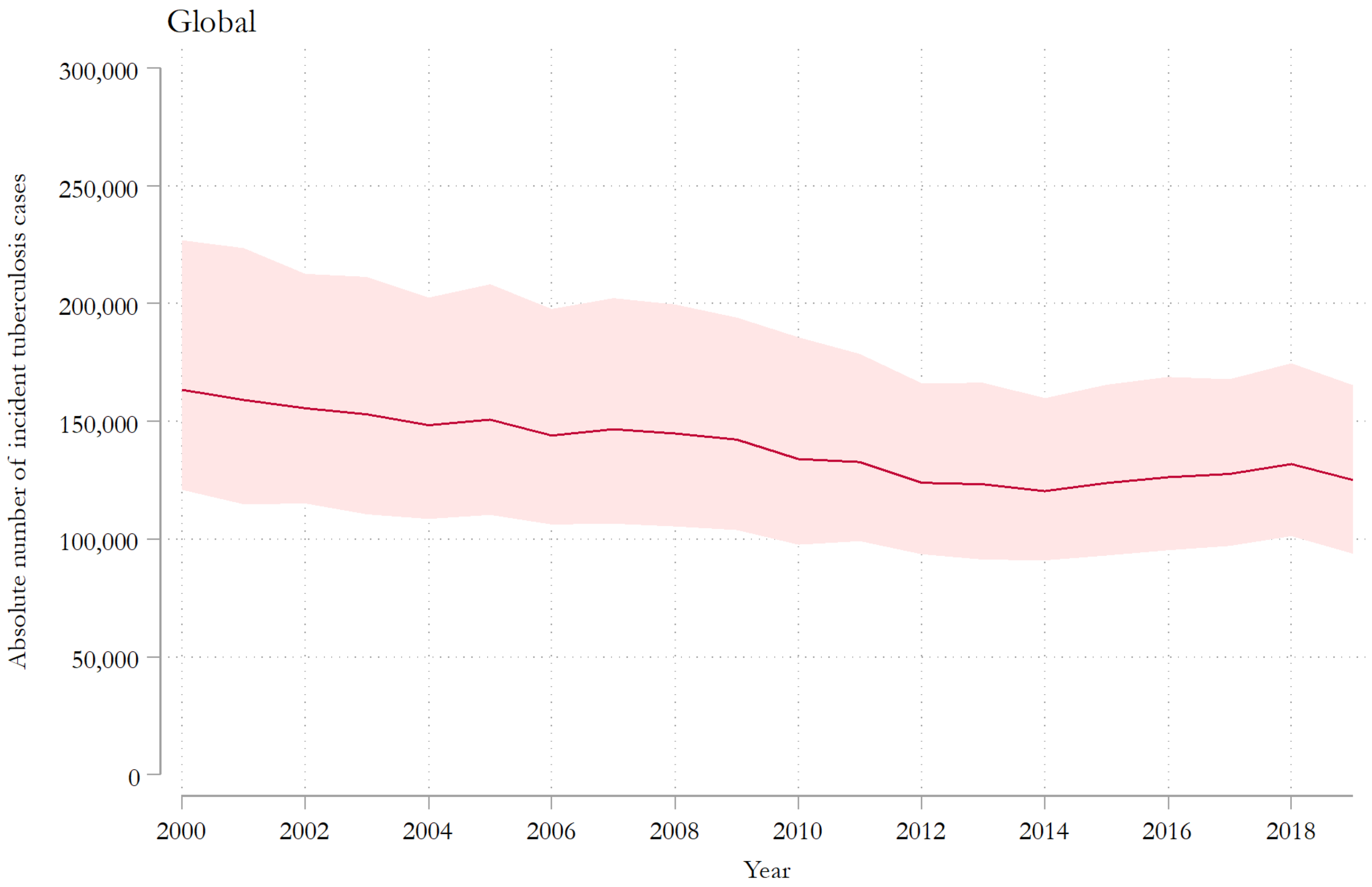

Figure S3. Global trends in the case detection ratio among incarcerated persons, 2000–2019.

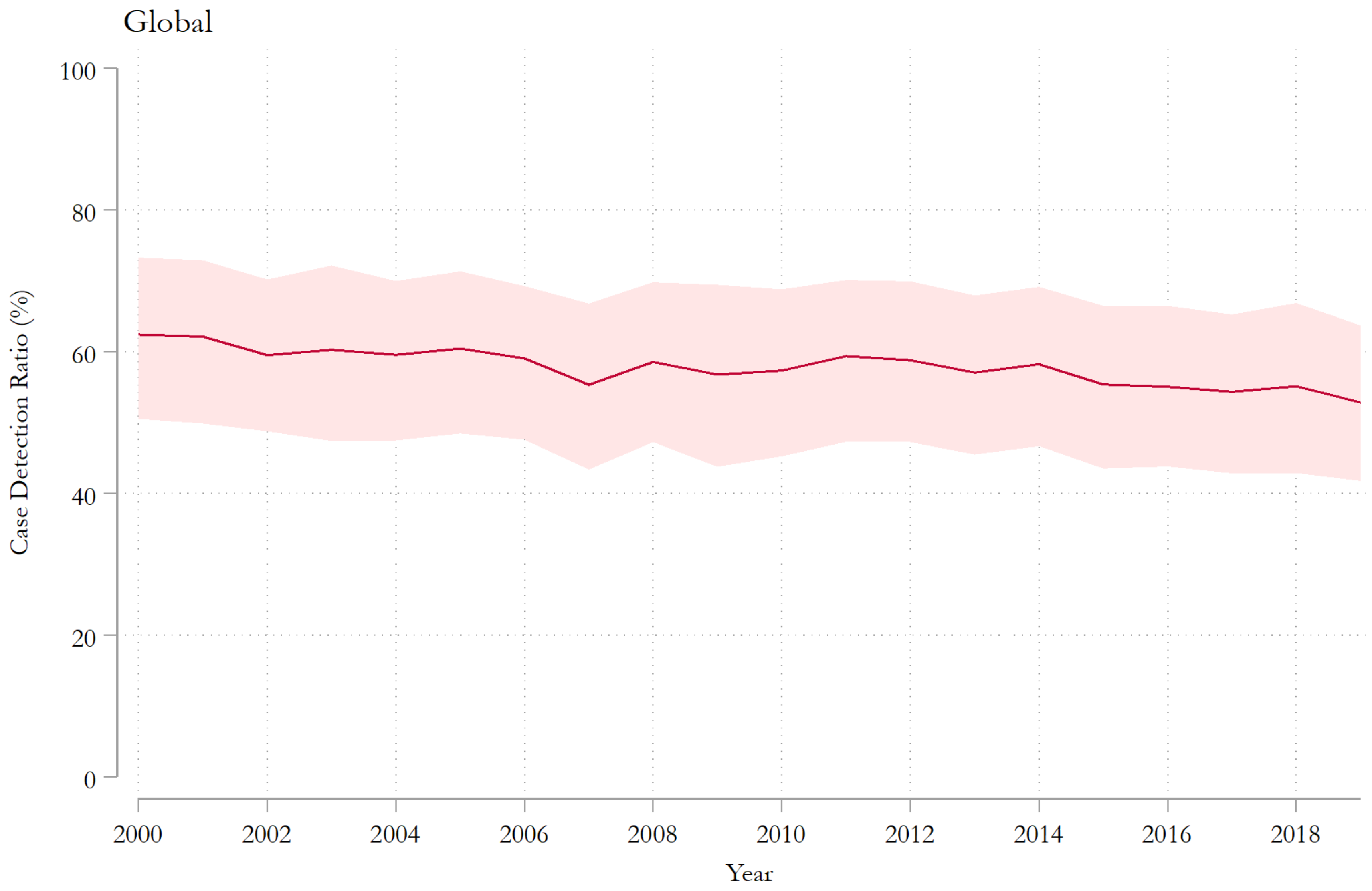

Solid red lines and shaded bands represent modelled point estimates (posterior medians) and 95% credible intervals for the case detection ratio globally.

Figure S5. Tuberculosis incidence (cases per 100 thousand person-years) among incarcerated persons in Central, South, and North America, 2000–2019

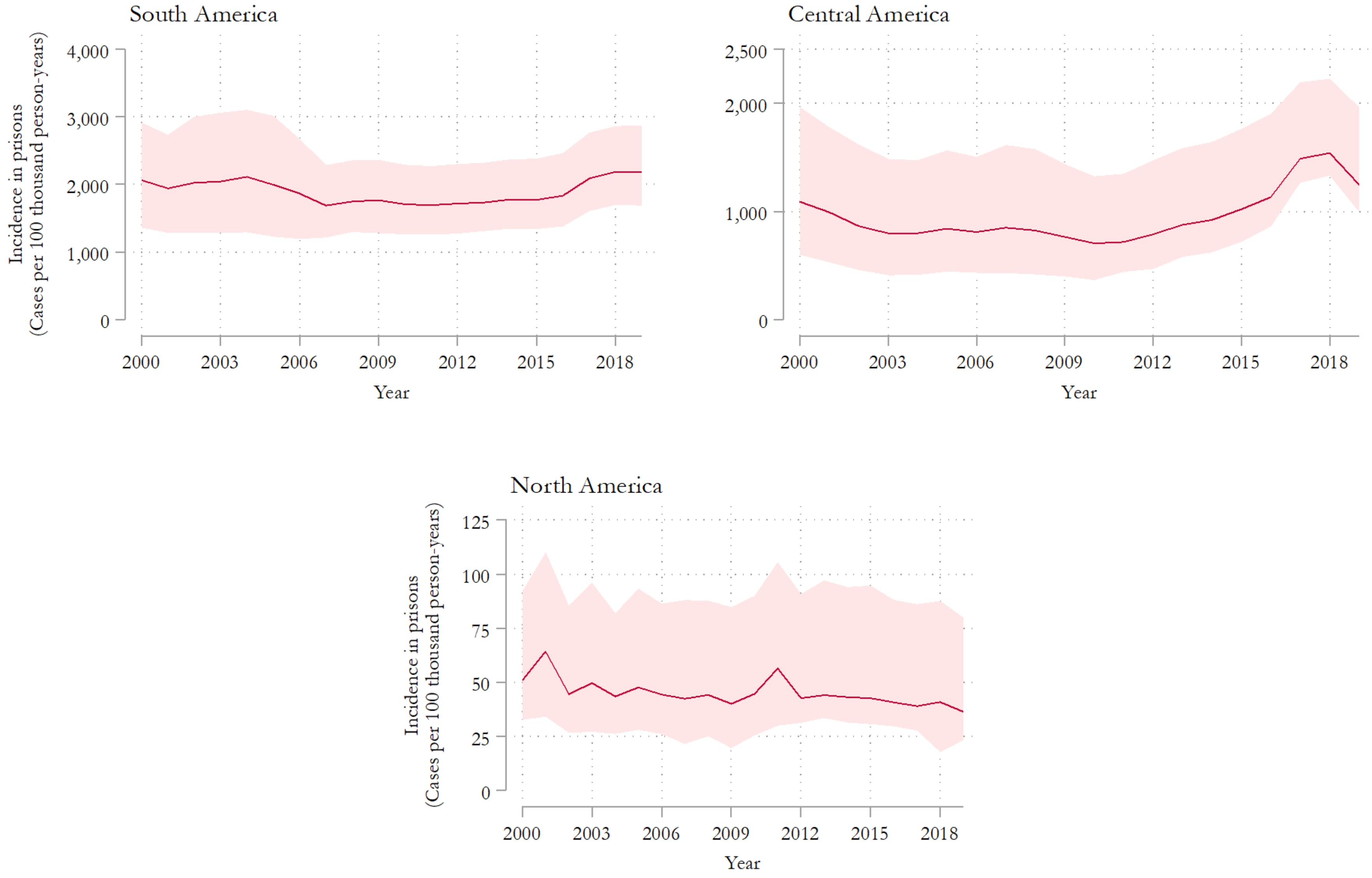

Figure S6. Tuberculosis data availability on tuberculosis prevalence and/or incidence from the systematic review and meta-analysis and on national tuberculosis notifications among incarcerated persons.

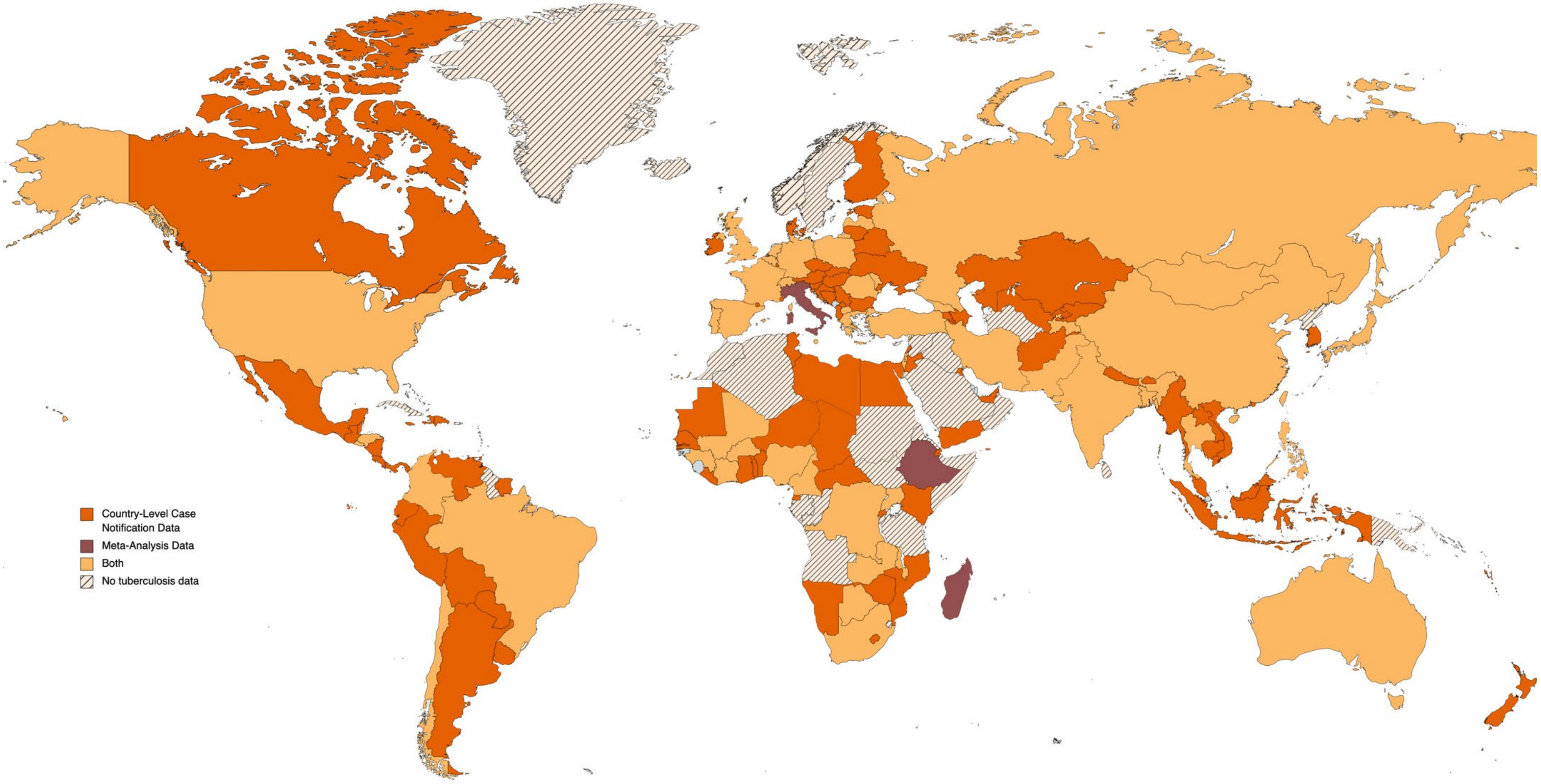

Figure S7. Data availability on tuberculosis prevalence and/or incidence from the systematic review and meta-analysis, national tuberculosis notifications among incarcerated persons, and incarceration data.

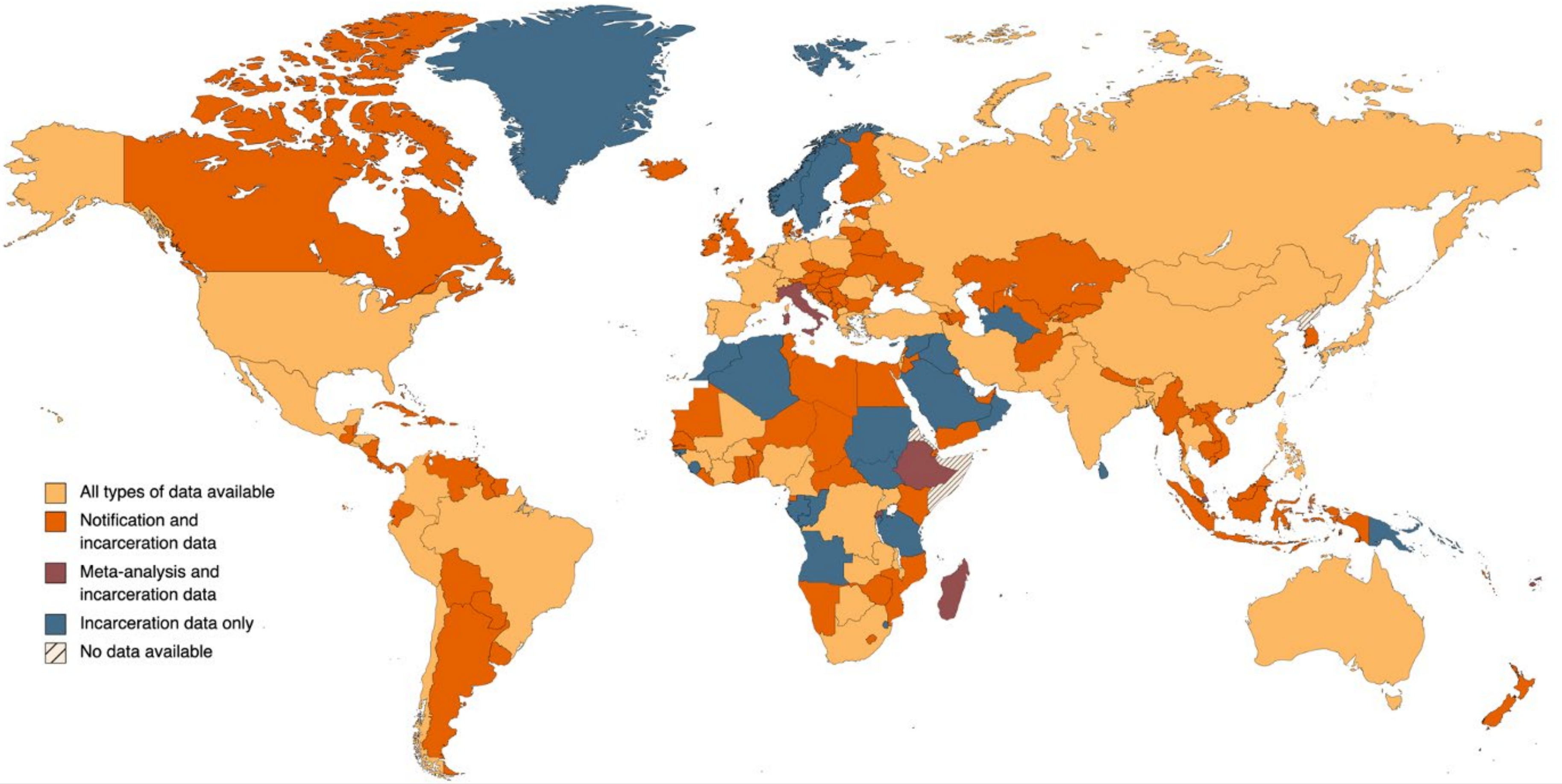

Figure S8. Numbers of new tuberculosis cases (dark grey) and tuberculosis notifications (light grey) in the high tuberculosis burden countries.

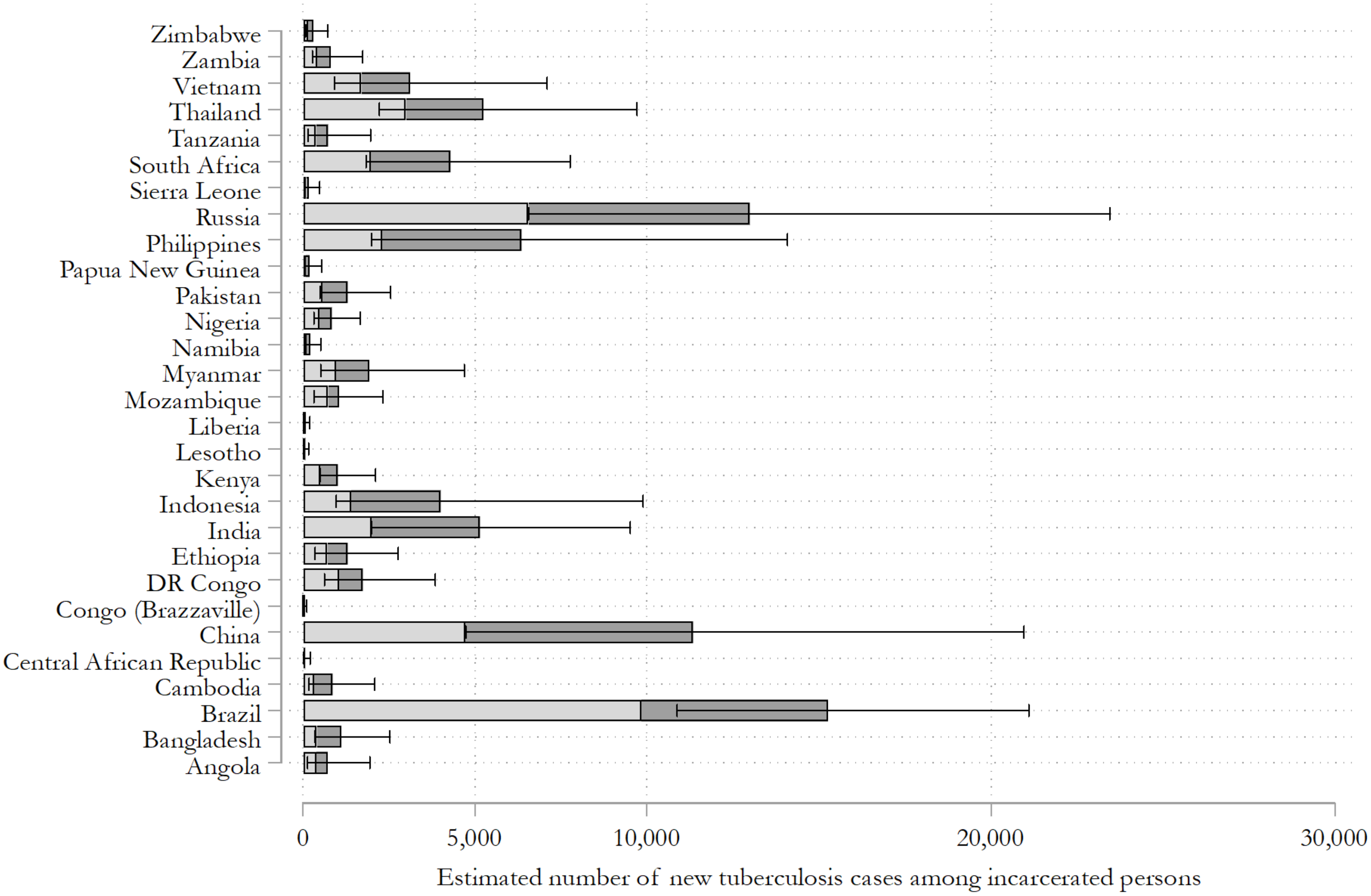

Figure S9. Tuberculosis incidence per 100 thousand person-years and the absolute number of tuberculosis cases in each World Health Organization region, 2019.a

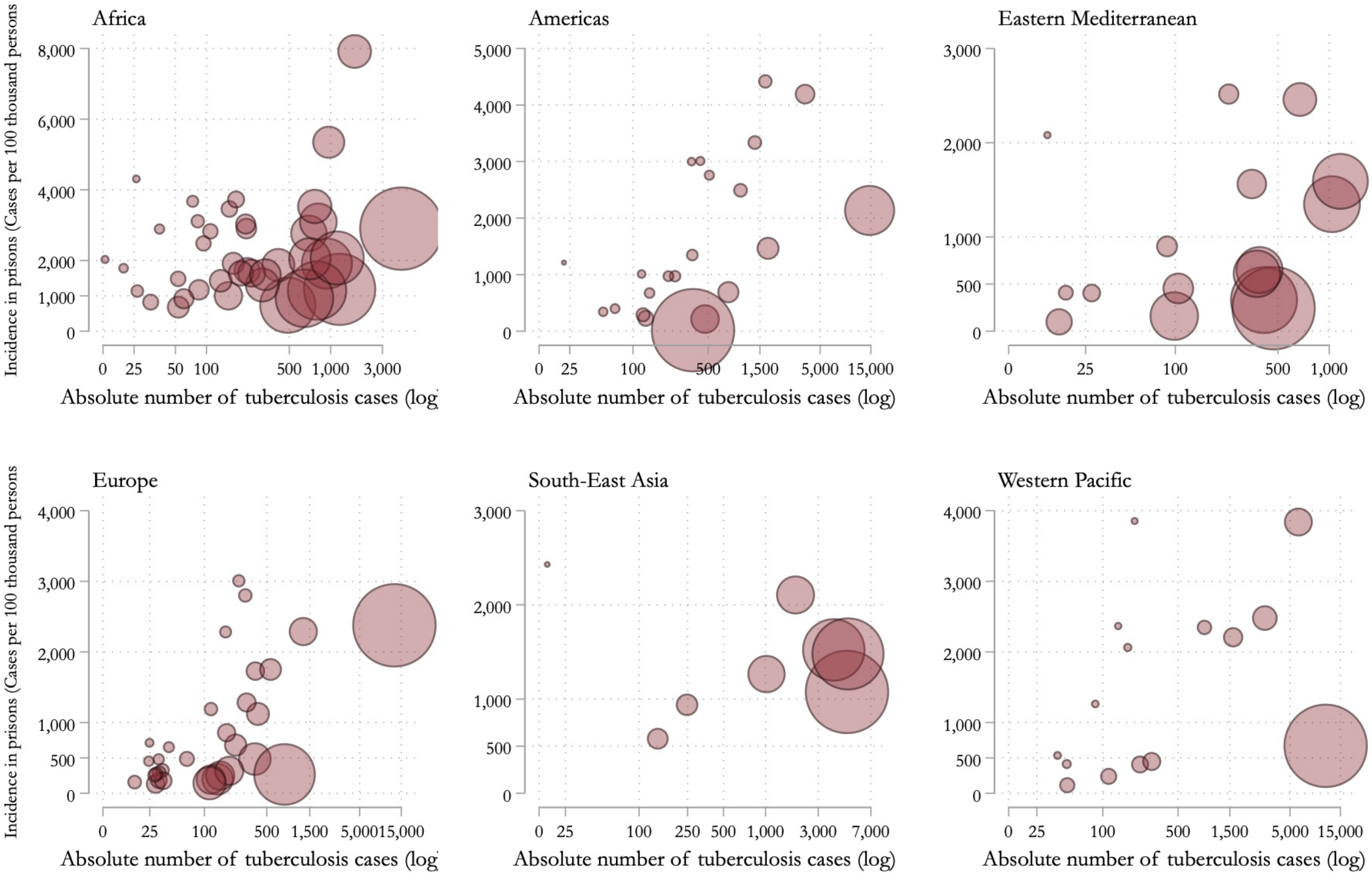

Figure S10. Predicted values (posterior medians) compared to observed tuberculosis incidence estimates from the validation dataset (log scale).

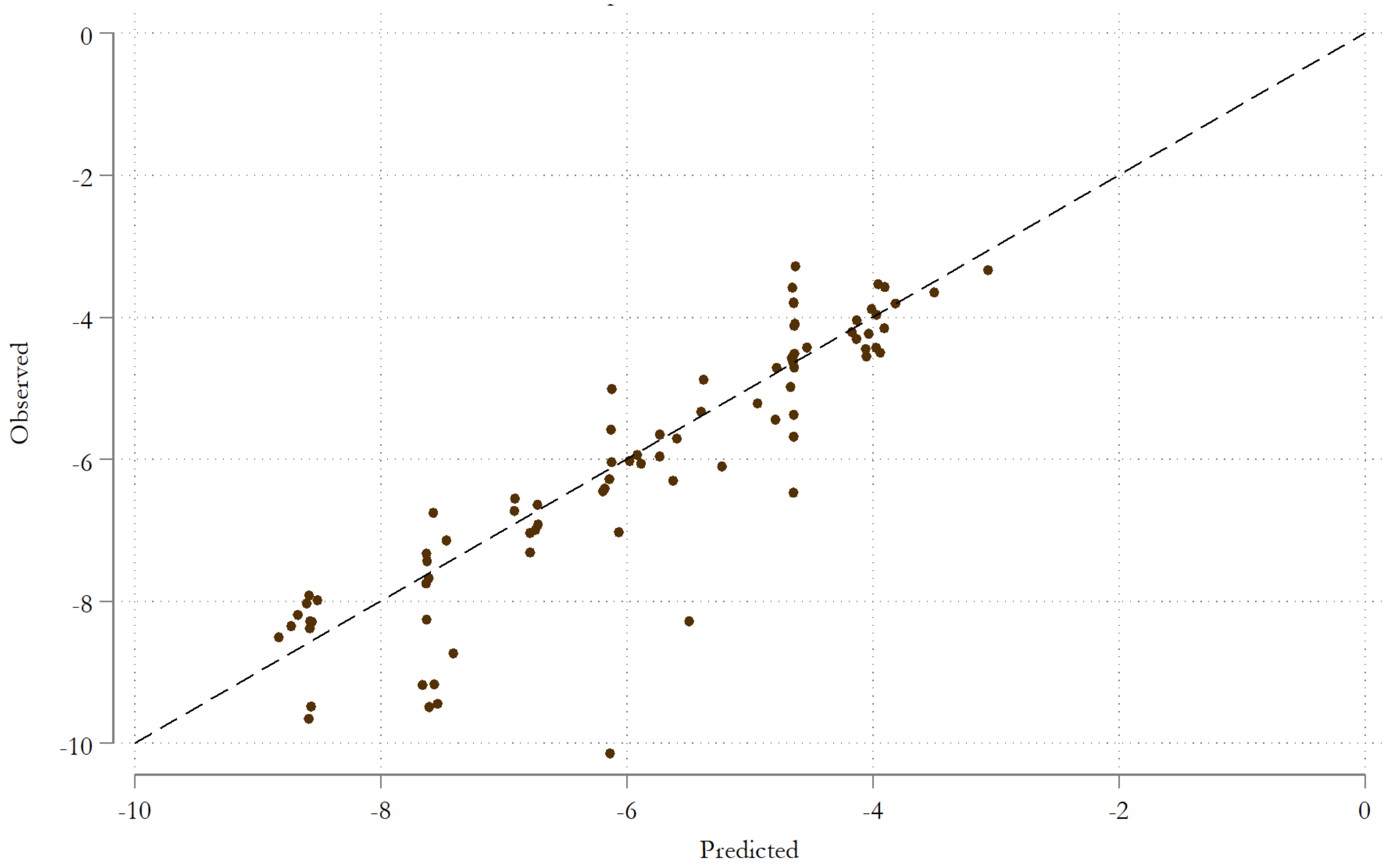

Figure S11. Predicted values (posterior medians) compared to observed tuberculosis notifications from the validation dataset (log scale).

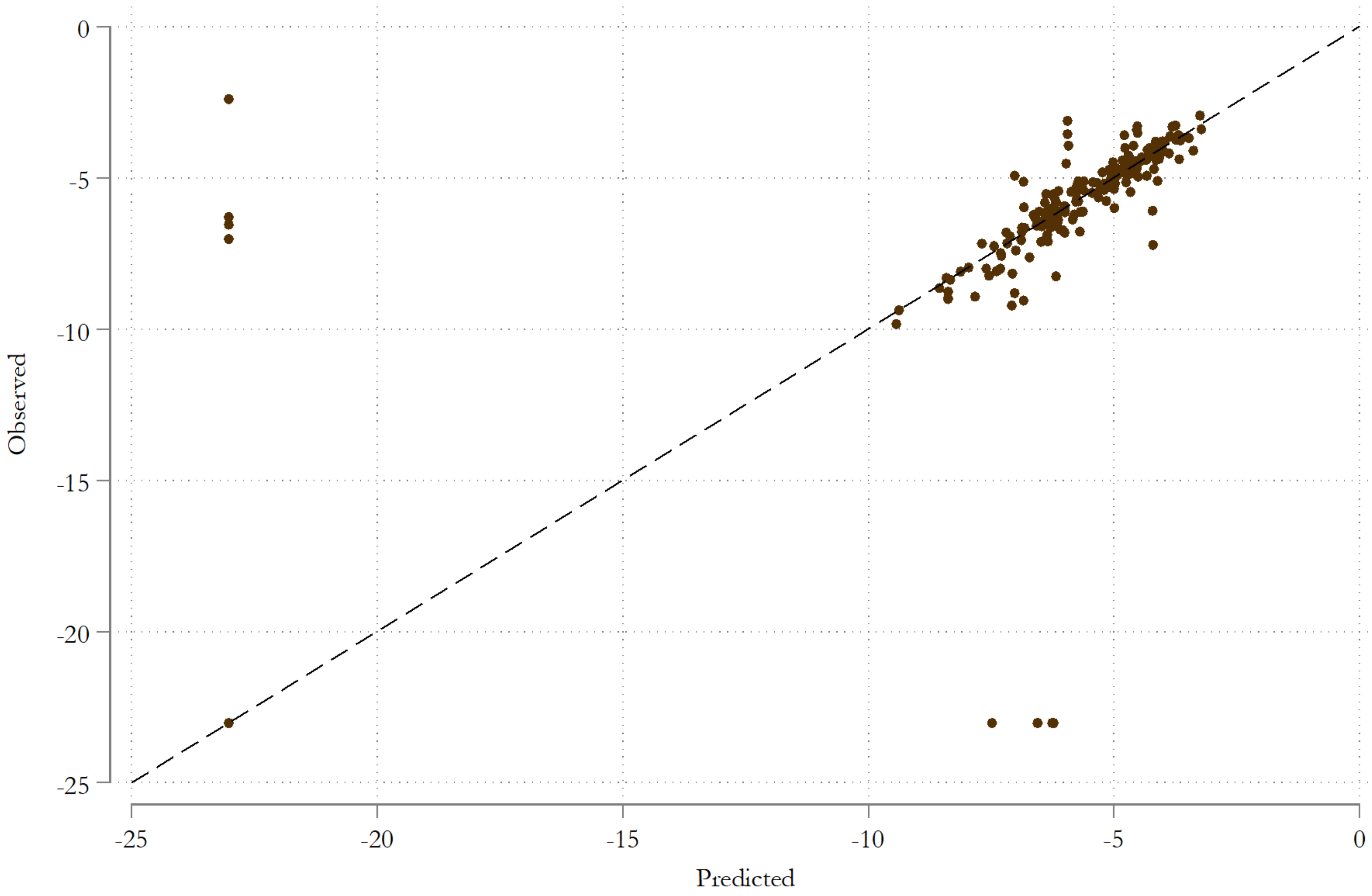

Figure S12. Predicted values (posterior medians) compared to observed tuberculosis prevalence estimates in the validation dataset (log scale).

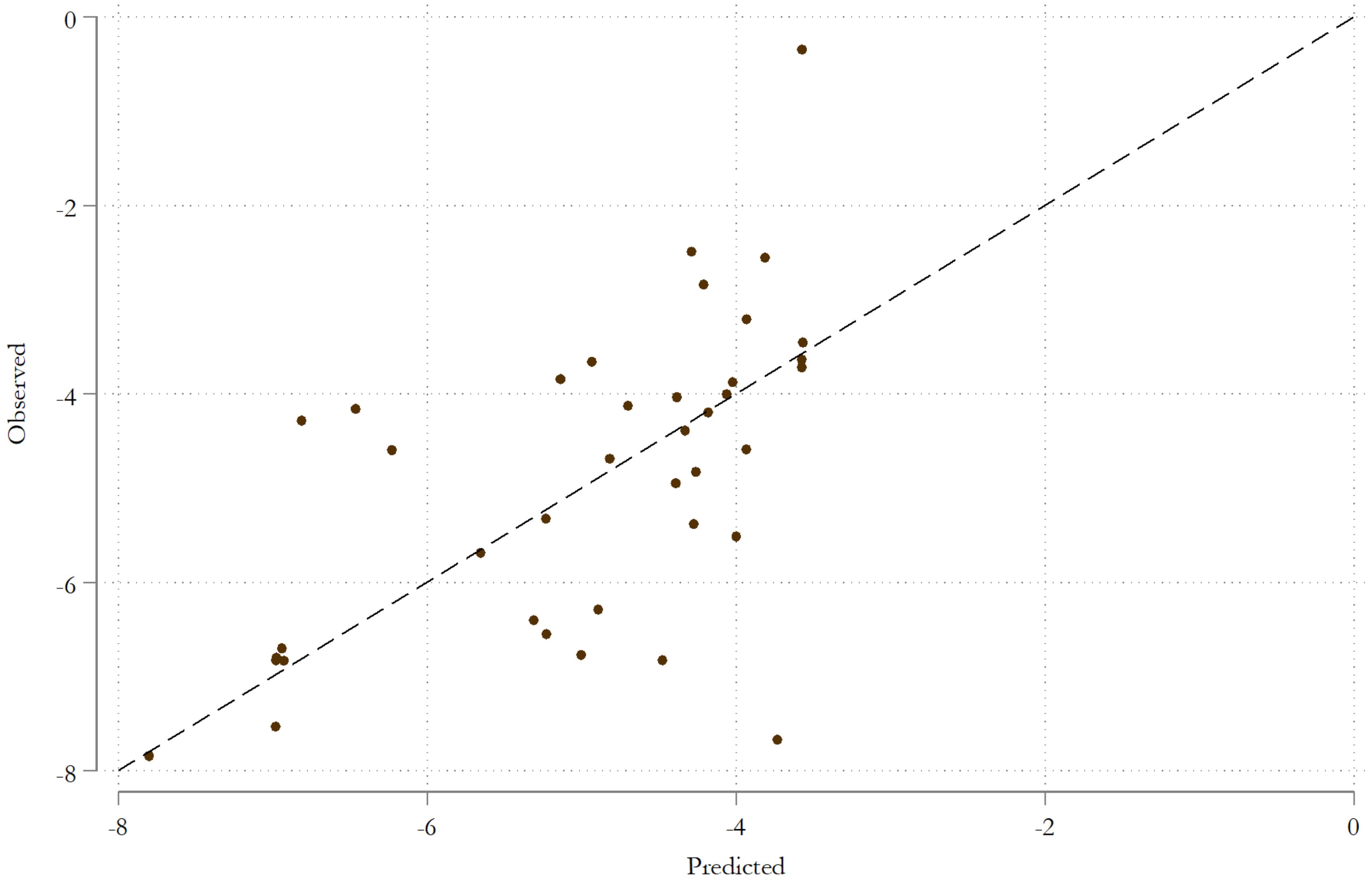

Figure S13. The relationship between estimated prison-level tuberculosis notifications (left hand panel) and incidence (right hand panel) with capacity in prisons.

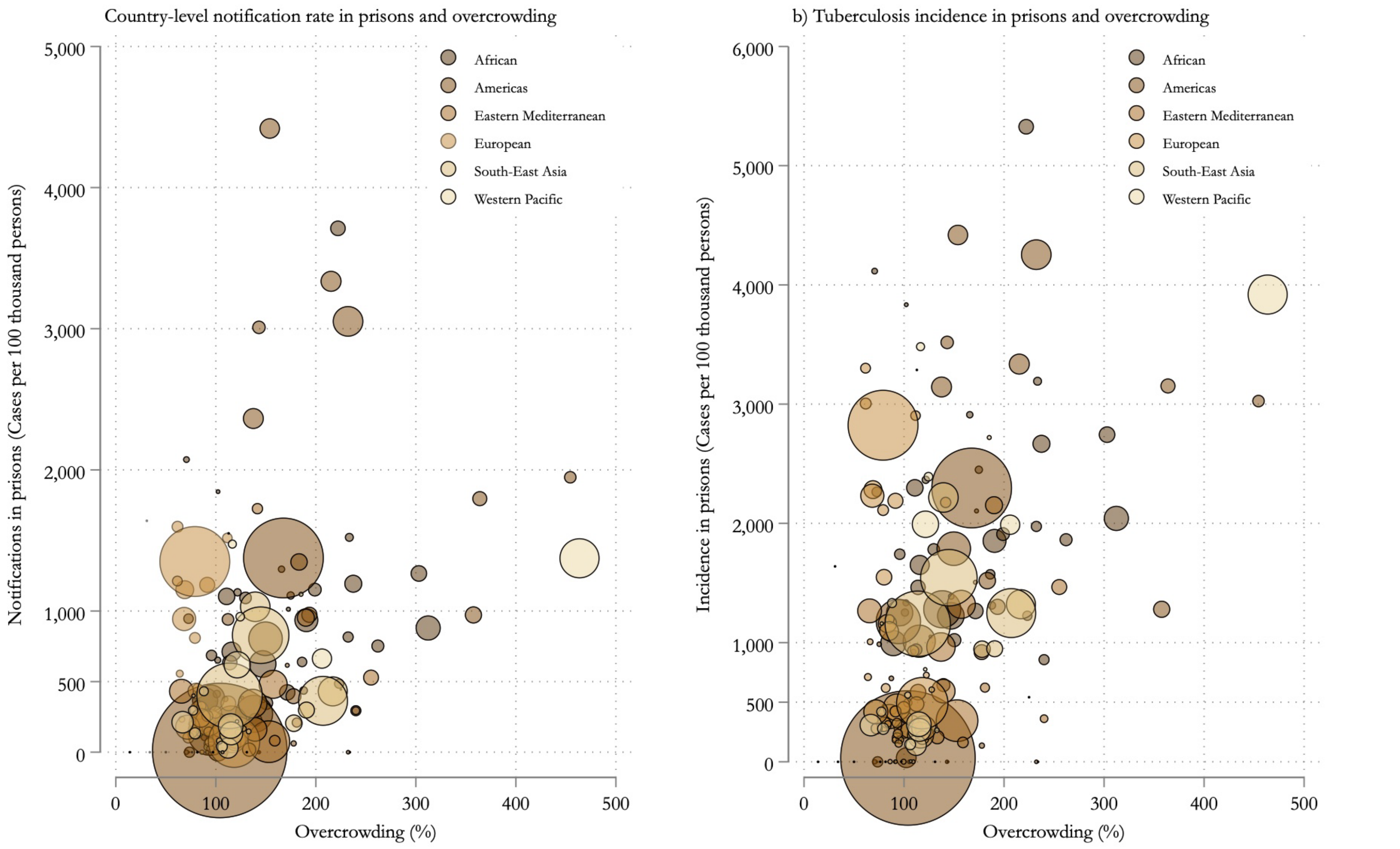

Each bubble represents a distinct country. Bubble size is weighted by the absolute number of incarcerated persons in that country. Therefore, countries with large populations of incarcerated persons are larger (i.e., the United States is the largest bubble displayed due to

their status as having the most incarcerated persons globally). Distinct shading of the bubbles represent differing World Health Organization regions as indicated by the legend. Linear fit prediction plots are displayed with corresponding 95% confidence intervals for each graph. These prediction plots and analyses were done post-hoc after country-level estimates were derived from the meta-regression model. Estimates of tuberculosis incidence in the general population were taken from World Health Organization estimates of country-level markers. Overcrowding is defined as the number of incarcerated persons in that country divided by the official capacity of the prison system. Figure 5a only includes estimates from 2019 (for tuberculosis incidence in both prisons and the general population), the most recent year of data. Figure 5b includes several years of data depending on the availability of overcrowding data. All overcrowding data are matched by year to tuberculosis incidence estimates in prisons.

Figure S14. Absolute number of incident tuberculosis cases in prisons and the general population over time, Eastern Mediterranean

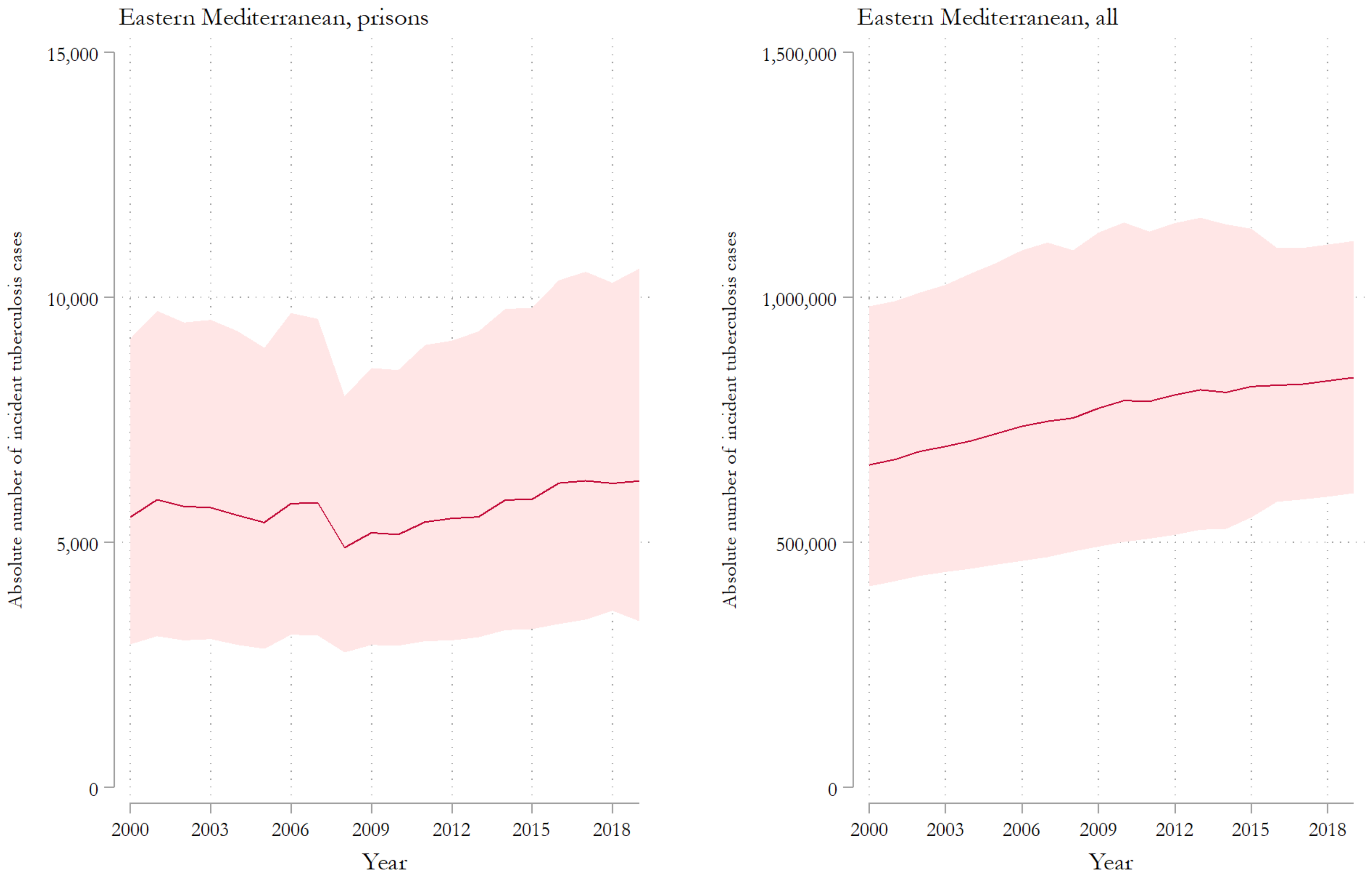

Figure S15. Absolute number of incident tuberculosis cases in prisons and the general population over time, Western Pacific.

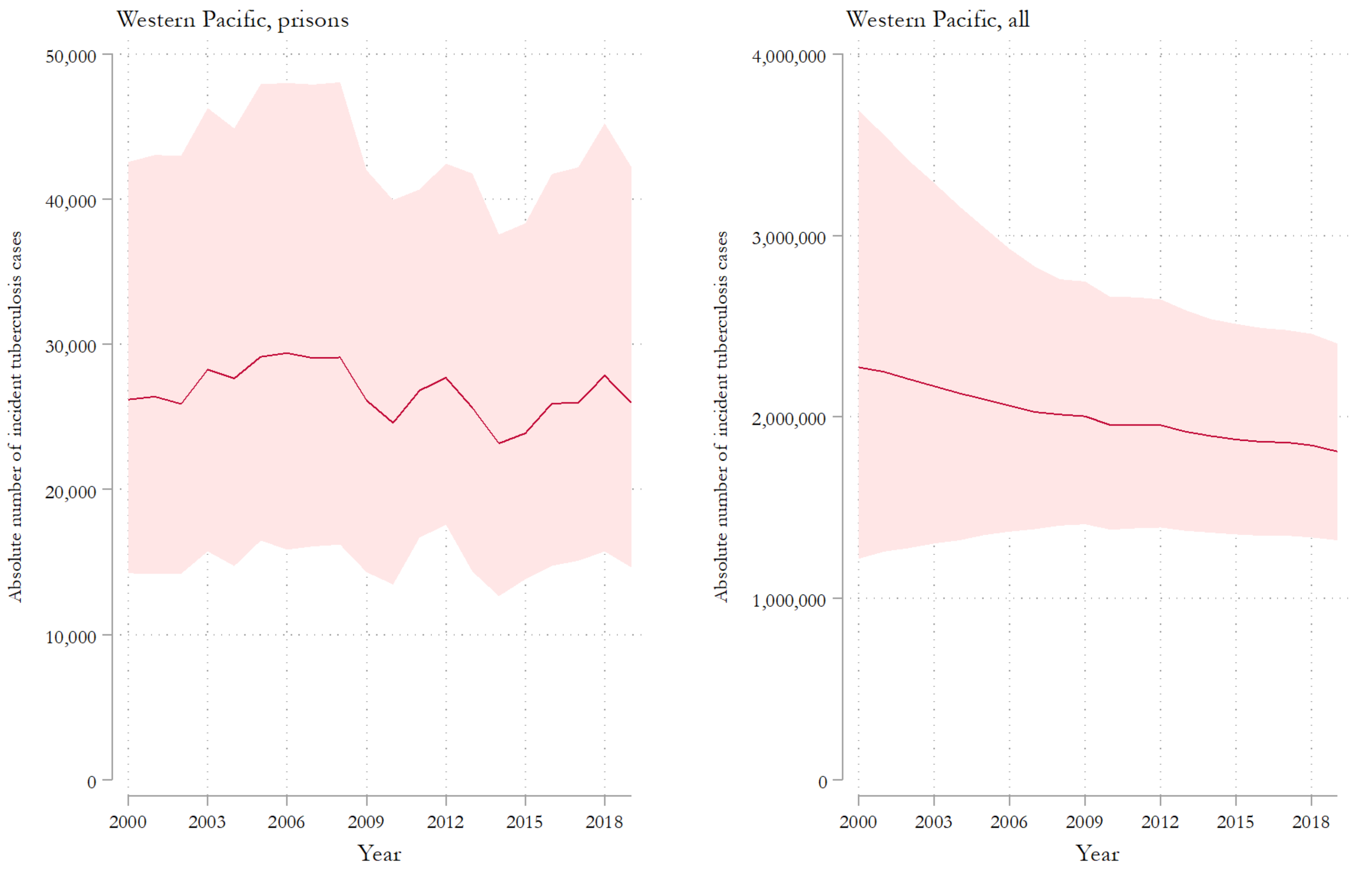

Figure S16. Absolute number of incident tuberculosis cases in prisons and the general population over time, South-East Asia.

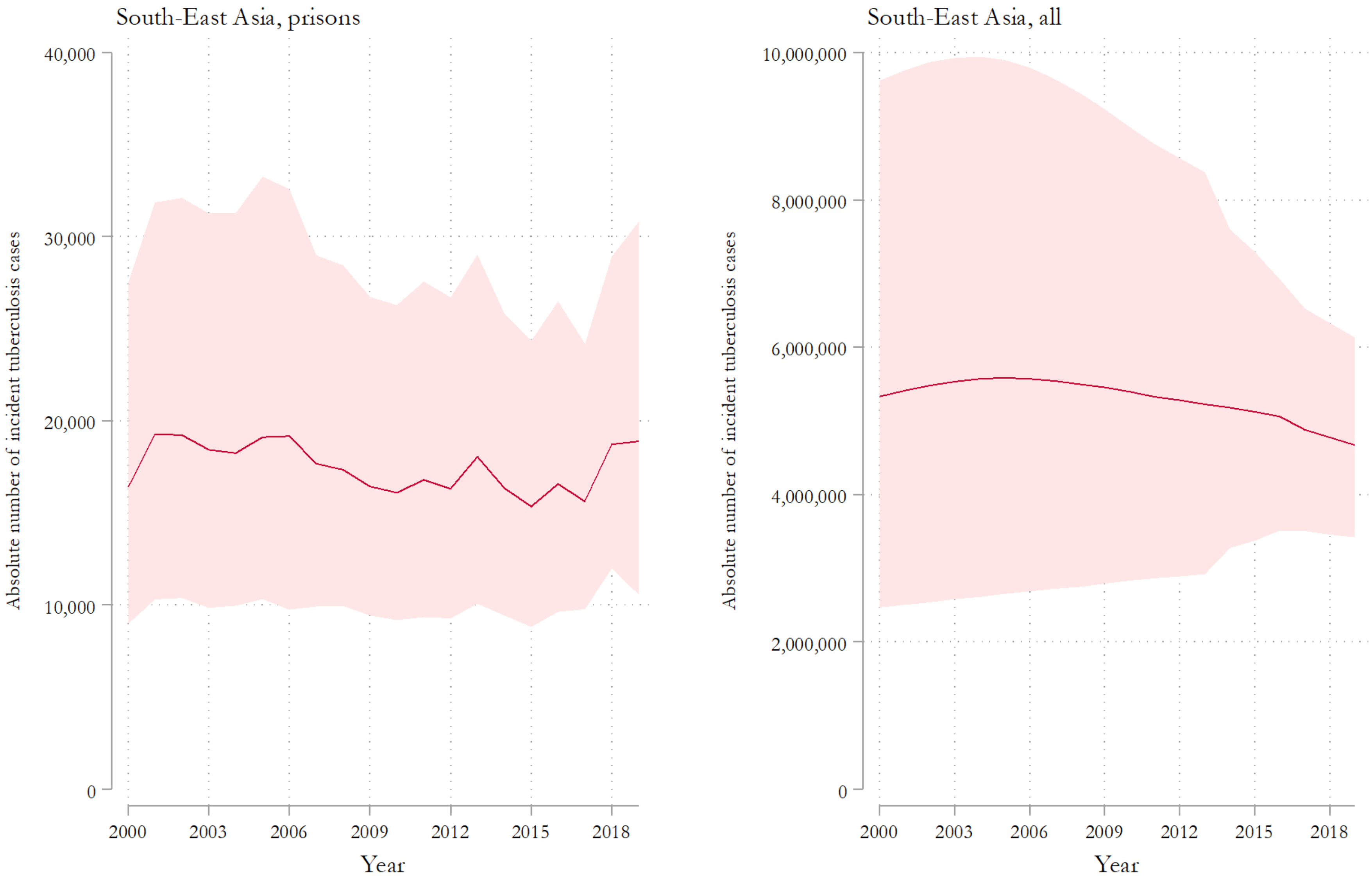

Figure S17. Absolute number of incident tuberculosis cases in prisons and the general population over time, Americas.

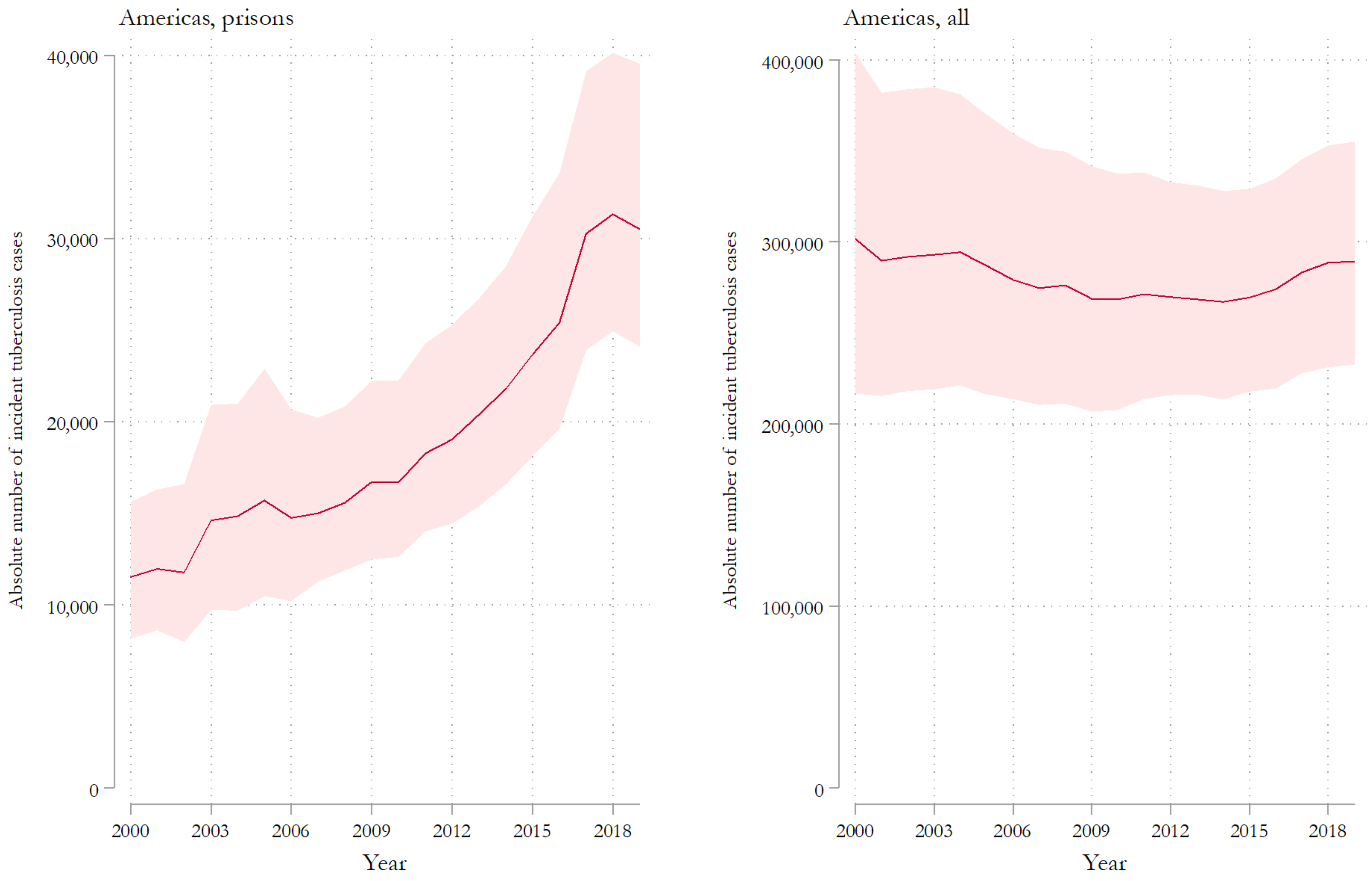

Figure S18. Absolute number of incident tuberculosis cases in prisons and the general population over time, Europe.

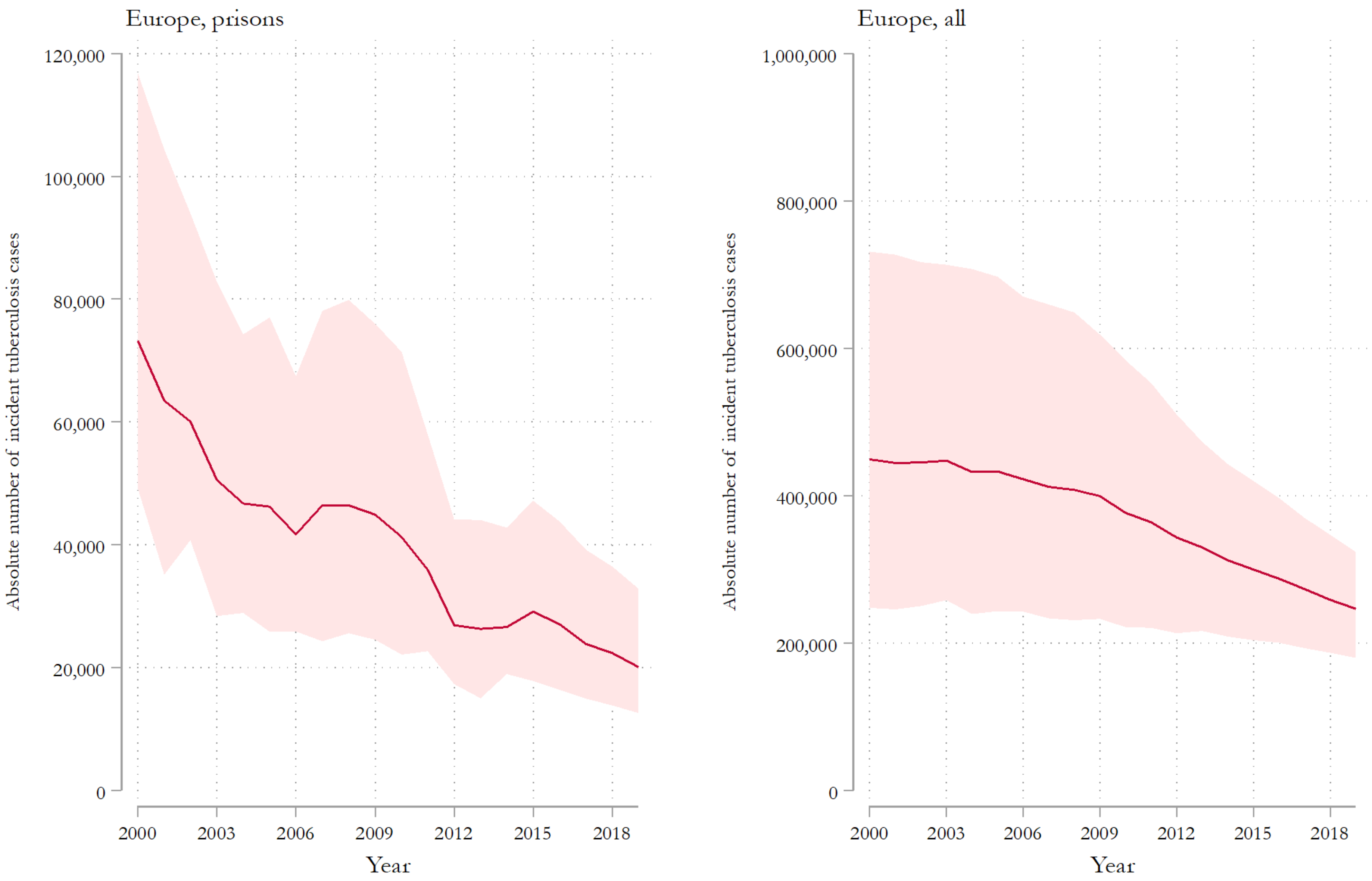

Supplement: Supplementary appendix [file mmc1.pdf]
